# Smooth Muscle-Targeted Overexpression of Peroxisome Proliferator Activated Receptor-γ Disrupts Vascular Wall Structure and Function

**DOI:** 10.1371/journal.pone.0139756

**Published:** 2015-10-09

**Authors:** Jennifer M. Kleinhenz, Tamara C. Murphy, Anastassia P. Pokutta-Paskaleva, Rudolph L. Gleason, Alicia N. Lyle, W. Robert Taylor, Mitsi A. Blount, Juan Cheng, Qinglin Yang, Roy L. Sutliff, C. Michael Hart

**Affiliations:** 1 Atlanta VA Medical Center, Decatur, GA, United States of America; 2 Emory University, Atlanta, GA, United States of America; 3 Georgia Institute of Technology, Atlanta, GA, United States of America; 4 University of Alabama at Birmingham, Birmingham, AL, United States of America; Univeristy of Glasgow, UNITED KINGDOM

## Abstract

Activation of the nuclear hormone receptor, PPARγ, with pharmacological agonists promotes a contractile vascular smooth muscle cell phenotype and reduces oxidative stress and cell proliferation, particularly under pathological conditions including vascular injury, restenosis, and atherosclerosis. However, pharmacological agonists activate both PPARγ-dependent and -independent mechanisms in multiple cell types confounding efforts to clarify the precise role of PPARγ in smooth muscle cell structure and function *in vivo*. We, therefore, designed and characterized a mouse model with smooth muscle cell-targeted PPARγ overexpression (smPPARγOE). Our results demonstrate that smPPARγOE attenuated contractile responses in aortic rings, increased aortic compliance, caused aortic dilatation, and reduced mean arterial pressure. Molecular characterization revealed that compared to littermate control mice, aortas from smPPARγOE mice expressed lower levels of contractile proteins and increased levels of adipocyte-specific transcripts. Morphological analysis demonstrated increased lipid deposition in the vascular media and in smooth muscle of extravascular tissues. *In vitro* adenoviral-mediated PPARγ overexpression in human aortic smooth muscle cells similarly increased adipocyte markers and lipid uptake. The findings demonstrate that smooth muscle PPARγ overexpression disrupts vascular wall structure and function, emphasizing that balanced PPARγ activity is essential for vascular smooth muscle homeostasis.

## Introduction

PPARγ is a ubiquitously expressed nuclear hormone receptor that plays a critical role in regulating glucose and lipid metabolism. A diverse spectrum of naturally occurring fatty acids and their metabolites bind to and activate PPARγ which heterodimerizes with the retinoid X receptor to stimulate transactivation and transrepression pathways. PPARγ is also the target of high affinity, synthetic thiazolidinedione pharmacological ligands, including rosiglitazone and pioglitazone, which are employed clinically to stimulate PPARγ and enhance insulin sensitivity in selected patients with type 2 diabetes [1, 2]. In addition to roles in metabolic regulation, PPARγ is expressed in vascular wall cells including endothelial and smooth muscle cells *in vitro* and *in vivo* [3] where it contributes to regulation of vascular function, cell proliferation, inflammation, and redox balance [4]. While clinical studies indicate that pioglitazone administration reduces the degree of vascular dysfunction in diabetic patients [5], concerns have been raised that rosiglitazone promotes vascular complications [6]. These findings have generated confusion regarding the role of PPARγ activation in the pathogenesis and treatment of vascular disease and suggest that drug- rather than class-specific effects of thiazolidinediones may determine the impact of PPARγ activation on vascular disease outcomes. In addition, thiazolidinediones cause undesired side effects such as weight gain, fluid retention, and osteoporosis [7]. Taken together, these considerations indicate that while pharmacological PPARγ targeting provides therapeutic potential in vascular disease, further study will be required to develop new PPARγ ligands that optimize benefits and minimize adverse side effects of these drugs.

The role of PPARγ in vascular biology is further complicated by evidence that thiazolidinediones exert biological effects in some systems that are independent of PPARγ activation [8], and their systemic administration activates PPARγ in extravascular compartments. Mounting evidence suggests that TZDs exert beneficial effects on non-diabetic vascular pathophysiology. For example, PPARγ was reduced in the vasculature of spontaneously hypertensive rats, and vascular smooth muscle cells derived from these animals displayed reduced smooth muscle contractile protein levels and enhanced proliferative and migratory behavior [9]. PPARγ overexpression in these cells rescued smooth muscle contractile protein expression and attenuated the proliferative smooth muscle cell phenotype. Activation of PPARγ with rosiglitazone *in vivo* also increased aortic contractile protein expression and attenuated aortic remodeling in spontaneously hypertensive rats [9].

Because global knockout of the PPARγ gene in mice causes embryonic lethality [10], previous studies have employed tissue-targeted PPARγ deletion or inhibition strategies to clarify how loss of PPARγ function modulates normal vascular physiology and responses to pathophysiological stimuli [4]. Studies inducing endothelial or smooth muscle cell-targeted PPARγ knockdown or expression of dominant negative PPARγ have confirmed that loss of PPARγ function contributes to complex vascular phenotypes *in vivo* [11, 12]. For example, compared to littermate control animals, smooth-muscle-specific PPARγ knockout (smPPARγKO) mice had comparable [13] or decreased [11] systemic arterial pressures. In addition, in these studies, vascular contractility responses to phenylephrine were impaired in femoral arteries [13] but normal in aortas from smPPARγKO mice [11]. smPPARγKO mice were more susceptible to abdominal aortic aneurysms due to increases in cathepsin S [14]. The generalized PPARγKO (rescued from embryonic lethality) had reduced systemic arterial pressure, impaired aortic contraction to phenylephrine, increased aortic relaxation to acetylcholine, but no change in endothelial nitric oxide synthase levels [15]. Mice with inhibition of smooth muscle PPARγ using dominant-negative constructs demonstrate increased systemic arterial pressure and reduced aortic contraction to phenylephrine [12, 16]. Collectively, these studies demonstrate that loss of constitutive PPARγ function in smooth muscle cells exerts significant effects on the vascular function and impairs vascular contractility.

While current evidence supports that loss of vascular smooth muscle PPARγ impairs normal vascular function, conclusions from corollary studies examining PPARγ gain of function are limited largely to studies of pharmacological PPARγ ligands. And, it is not clear if the ultimate vascular effects of PPARγ ligands are mediated by systemic PPARγ activation, by direct stimulation of PPARγ in vascular wall cells, or both. Because PPARγ activation with TZDs reduces vascular dysfunction in a variety of pathophysiologically relevant models, and because smooth-muscle-PPARγ-deletion generally perturbs normal vascular function, we hypothesized that overexpression of PPARγ in smooth muscle would have a beneficial impact on vascular function. To test this hypothesis, we created a novel transgenic mouse model with inducible and targeted overexpression of constitutively active PPARγ in smooth muscle cells. Our findings demonstrate that robust overexpression and activation of smooth muscle cell PPARγ stimulates significant derangements in vascular structure and function. The mechanisms underlying these derangements were related to PPARγ-induced smooth muscle cell-to-adipocyte transdifferentiation. Combined with previous reports, these results demonstrate that either loss or gain of vascular smooth muscle PPARγ activity is sufficient to cause vascular derangements indicating that balanced PPARγ activity in vascular smooth muscle is required for normal vascular function.

## Materials & Methods

### Generation of Smooth Muscle-Targeted PPARγ Over-Expressing Mice

The transgenic mouse model with smooth muscle-specific overexpression of a constitutively active mutant PPARγ gene (VP16-PPARγ) was generated in C57Bl/6J mice using a strategy similar to that used to generate a transgenic line with tissue-specific overexpression of PPARδ[17, 18]. The VP16-PPARγ fusion protein is a constitutively active form of PPARγ with activity similar to the natural receptor in the presence of ligand [19–22]. The transgenic line (in the C57Bl/6J strain) of VP16-PPARγ contains a floxed stop sequence (CAT) driven by the human cytomegalovirus immediate early enhancer/chicken β-actin (CAG) promoter. These mice were crossed with mice (in the C57Bl/6J strain) expressing Cre recombinase driven by the smooth muscle myosin heavy chain (SMMHC) promoter [23]. Expression of Cre recombinase removes the CAT stop sequence and results in the selective overexpression of VP16-PPARγ in smooth muscle cells. DNA isolated from tail snips of offspring from these crosses were genotyped and littermate control (floxVP16-PPARγ -/-; SMMHC Cre X/Y^+^) and smPPARγOE mice (floxVP16-PPARγ +/-; smmhcCre X/Y^+^) were selected for additional study. Because the SMMHC Cre transgene is inserted into the Y chromosome, only male animals were examined in this study. At 6 weeks of age, littermate control and smPPARγOE mice were injected with tamoxifen (50 mg/kg/day by IP injection for 5 days). Unless stated otherwise, mice were euthanized 4–10 weeks later for experiments. This strategy not only permitted PPARγ transgene expression and activation in a vascular smooth muscle cell-specific fashion, but the inducible Cre-recombinase expression permitted induction of PPARγ once the vasculature had matured thereby avoiding potential developmental effects of transgene expression. The expression of this Cre recombinase in a smooth muscle-specific manner was previously confirmed by cross-breeding with ROSA reporter mice [23]. All studies were performed according to protocols reviewed and approved by the Atlanta VA Medical Center Animal Care and Use Committee (Protocol #V003-14). All animal procedures conform to NIH guidelines (*Guide for the care and use of laboratory animals*). Surgeries were performed under inhalational isoflurane, and all efforts were made to minimize suffering. Euthanasia was performed with CO_2_ overdose.

### General Phenotyping

Littermate control and smPPARγOE mice were subjected to a broad variety of studies to establish the impact of smooth muscle-targeted PPARγ activation on phenotype. Food and water consumption were measured over 24 hours in individually-housed mice. Glucose tolerance tests were performed by injecting mice with intraperitoneal glucose (2 g/kg body weight) then determining glucose concentration of blood obtained from the tail vein at 0, 20, 40, 60, 120, and 240 minutes using an Accu-Chek Avia meter. Urine osmolality was measured on a Wescor 5520 Vapor Pressure Osmometer (Wescor, Logan, UT). Urinary sodium, chloride, and potassium were measured by the EasyLyte (Medica, Bedford, MA) instrument. Urine creatinine was determined with a kit by the *Jaffe* reaction method (BioVision, Milpitas, CA). Metabolic rates were measured on individual mice with the Oxymax Lab Animal Monitoring System (Columbus Instruments, Columbus, OH).

### RNA Isolation and Quantitative RT-PCR

After euthanasia with CO_2_ overdose, thoracic aortas were isolated, and the adventitial layer was removed. The remaining tissue was ground into a powder using a mortar and pestle in liquid nitrogen. Total RNA was isolated with TRIzol (Life Technologies, Grand Island, NY). Quantitative RT-PCR was performed with iScript One-Step RT-PCR Kit with SYBR Green (Bio-Rad) and the 7500 Fast Real-Time PCR system (Life Technologies) using primer sequences detailed in S1 Table. Amplicon expression in each sample was normalized to levels of ribosomal 9s RNA. The relative abundance of target mRNA in each sample was calculated using ΔΔCT methods and expressed as fold change (2^-ΔΔCT^) [24].

### Western Blotting

Mouse aortic homogenates were suspended in lysis buffer (20 mM Tris pH 7.4, 2.5 mM EDTA, 1% Triton X-100, 1% deoxycholic acid, 0.1% sodium dodecyl sulfate, 100 mM NaCl, 10 mM NaF, 1 mM Na_3_VO_4_) containing protease and phosphatase inhibitors (Complete Mini, EDTA-free and PhosSTOP, Roche). These suspensions were then sonicated and clarified by centrifugation. Protein (20 or 40 μg) was loaded per lane and separated by SDS-PAGE. Immunoblotting was performed with antibodies directed against ACTA1 (Thermo Fisher RB-9010-P, rabbit polyclonal, 1:500), CALD1 (Sigma C4562, mouse monoclonal, 1:1000), CNN1 (Sigma C2687, mouse monoclonal, 1:5000), PPARγ (custom rabbit polyclonal raised against peptide CEKTQLYNRPHEEPSNS, 1:2000), and CDK4 (Santa Cruz sc-260, rabbit polyclonal, 1:500) as reported [25]. Relative levels of immunoreactive proteins were quantified using the ChemiDoc XRS imaging system and Quantity One software (Bio-Rad).

### PPARγ Activity Assay

Nuclear extracts of selected samples were prepared in complete lysis buffer using a nuclear extraction kit (Active Motif, Carlsbad, CA). PPARγ activity was then quantified using the TransAM PPARγ activity kit (Active Motif) which employs an ELISA-based immobilized oligonucleotide containing PPARγ response elements. A primary antibody recognizes accessible epitopes on PPARγ protein upon DNA binding. A secondary HRP-conjugated antibody is added, and colorimetric readouts are obtained using spectrophotometry to estimate relative differences in PPARγ nuclear binding.

### Blood Pressure Monitoring

Blood pressures in awake littermate control and smPPARγOE mice were measured by telemetry as previously reported [26] using sterile PA-C10 blood pressure probes (Data Sciences International, St. Paul, MN). Mice were induced with 4% isoflurane, and anesthesia was maintained for the duration of the procedure using 1.5–2% isoflurane. The anterior neck was shaved and disinfected with 70% alcohol and Betadine solution. Atropine (0.25 mg/kg) was injected subcutaneously to minimize airway secretions, and the mouse was covered with a sterile surgical drape. A ventral midline incision was made from the lower mandible to the sternum. The left common carotid artery was isolated, and sutures were passed under the carotid and used for both ligation and retraction. The anterior suture was tied off just caudal to the carotid bifurcation, and the posterior suture was placed as far caudal as possible. A 25-gauge needle with a bend at the beveled tip was used to hold open the carotid while the catheter was advanced into the thoracic aorta. The catheter was secured to the carotid. The transmitter battery was placed in a subcutaneous pouch along the right flank close to the hindlimb formed using blunt dissection. The neck incision was closed with suture. One cc of warmed Ringers’ lactate and buprenorphine (0.05 mg/kg) were administered post-operatively. Buprenorphine (0.05mg/kg, twice daily) was administered for 3 days post-operatively to alleviate discomfort related to the procedure. The mice were singly caged and allowed to fully recover for 7 days prior to the initiation of data collection. Blood pressures were then monitored for 10 s each minute for 24-h periods.

### *Ex Vivo* Aortic Ring Contractility Studies

Isometric vascular contractile forces were measured in aortic rings using a Harvard Apparatus differential capacitor force transducer (Holliston, MA) as described previously [26]. The resting tension of each ring was set to 20 mN and maintained throughout the experiment. Contractile responses to graded concentrations of KCl and L-phenylephrine were recorded. Data were obtained using Powerlab hardware and analyzed with LabChart software (AD Instruments, Colorado Springs, CO).

### Vascular Compliance

Cylindrical biaxial biomechanical tests of littermate control and transgenic aortas were performed as described previously [27]. After excision, aortas were cleaned of loose perivascular tissue and maintained in Dulbecco’s Phosphate-Buffered Saline (DPBS). Intercostal aortic branches were ligated with suture, and thoracic segments (4–6 mm long) were mounted onto glass cannulae (1.2 mm outer diameter) attached to a custom-made testing device as previously described [28] with minor modifications. Aortas were suspended in a DPBS bath containing sodium nitroprusside (SNP) to ensure complete dilation. Vessels were preconditioned with an inflation/deflation cycle (0-160-0 mm Hg) for 6 different levels of axial stretch (λ = 1.4, 1.45, 1.5, 1.6, 1.7, and 1.8). Subsequently, fixed stretch pressure-diameter tests were performed under quasi-static conditions for axial stretch levels λ = 1.4, 1.45, 1.5, 1.6, 1.7, and 1.8 for three pressurization cycles (0–160 mm Hg). Outer diameter data were collected from a thoracic aortic segment located between the 6^th^ and 7^th^ intercostal branch away from the celiac artery. The data from the third pressurization cycle at each stretch level was used for analysis. Fixed pressure force-length tests concluded the biomechanical testing procedure where the aortas were subjected to three axial loading cycles (0–3 g) while maintaining constant pressure levels (0, 40, 60, and 80 mm Hg), and the data of the last cycle at each pressure was used for analysis. *In vivo* axial stretch was determined as the intersection of the averaged force-length curves at a given pressure level [27]. Peterson’s modulus was calculated as the ratio of the pressure increment ΔP over the outer diameter change ΔD at a given pressure, normalized to the outer diameter at that pressure, E_p_ = ΔP/(ΔD/D). Arterial compliance was calculated as the inverse of the Peterson’s modulus, a standard measure of arterial stiffness (Compliance = 1/ E_p_).

### Histology and Morphometric Analysis

Aortas were perfused at constant pressure with phosphate buffered saline + 1 mM EDTA. The heart and 3 cm of aorta were then removed, cleaned, placed in formalin, and processed into paraffin blocks. Paraffin sections (5 μm) of the thoracic aorta were deparaffinized, fixed, and stained with H&E, Mason’s Trichrome, Accustain Elastic Stain (Sigma-Aldrich, St. Louis, MO), or Picro-sirius Red Stain (ScyTech, Logan, Utah) kits. In selected studies, frozen sections from the aorta were stained with Oil Red O (American Mastertech).

### MicroCT Imaging

Quantitative micro-computed tomography (CT) was used to generate CT angiograms of the vessels present in littermate control and smPPARγOE mice as described previously [29–31]. Mice were euthanized and sequentially perfused via left ventricular cannulation with saline containing 4 mg/mL of papaverine, followed by 0.9% normal saline, 10% neutral buffered formalin, and again with 0.9% normal saline. Animals were then perfused with Microfil contrast agent prepared per the manufacturer’s (Flow Tech, Carver, MA) protocol. Mice were decapitated, and the remaining body was immersed for 48 hours in 10% formalin. The bones of each corpse were de-mineralized in a formic acid based solution (Cal-Ex II, Fisher) for 21 days (replenishing with fresh Cal-Ex II every 7 days). Animals were then imaged at a 50 μm voxel size, and the tomograms were used to render binarized 3-D images. Using the vessel centerlines calculated in the Vascular Modeling Toolkit and a discretization of the reconstructed vessels into a 3-dimensional point array, vascular diameters were calculated continuously along the aorta. The centerline was fit to a quintic B Spline, and the first order derivative was employed along with the centerline point coordinates to define a cross sectional plane at each point of the centerline. The points of the reconstructed vessel that fell within one half of the original CT image voxel's inplane resolution at each point were then calculated, and these surface points were considered to be a part of the cross section. The distance from the centerline to each of these surface points was calculated, and each set was averaged to yield an "average radial distance" resolved along the length of the centerline.

### Echocardiography

Echocardiography studies were completed as described previously [32]. Mice were anesthetized with 4% isoflurane, hair was removed from the thorax, and mice were maintained under light anesthesia (1–1.5% isoflurane). Two-dimensional and M-mode transthoracic echocardiography modalities were used to assess wall motion, chamber dimensions, and wall thickness and to calculate percent fractional shortening (FS), stroke volume (SV), and ejection fraction (EF). For each measurement, at least 3 beats were averaged per measurement, at least 3 measurements were taken per animal, and beats were taken at end expiration. Studies were reviewed by two different investigators, one of whom was blinded. Measurements were made using a VisualSonics® Vevo 770TM *in-vivo* micro-imaging system equipped with a RMV-707B cardiovascular scanhead (Toronto, ON).

### Cell Culture

Human aortic smooth muscle cells (hAoSMC) were purchased directly from Lonza (Basel, Switzerland), Catalog # CC-2571. hAoSMC monolayers were grown at 37°C in a 5% CO_2_ atmosphere in culture media (SmGM-2, Lonza) containing 5% fetal calf serum, growth factors, and antibiotics. Human PPARγ in adenovirus (Ad-hPPARγ) or Ad-GFP (Vector Biolabs, Philadelphia, PA) were applied to cells at 25 MOI for 5 hours in 2% FBS media. Media were exchanged with fresh SmGM-2 media, and hAoSMC were cultured for an additional 3–7 days. In selected studies, hAoSMC were treated with 10 μM rosiglitazone after transfection.

### Statistical Analysis

All data are expressed as mean ± SEM. All analyses were performed using Graph-Pad Prism v. 6.03 (GraphPad Software, San Diego, CA). Statistical methods employed in each study are indicated in the figure legend. A *P* value <0.05 was considered significant.

## Results

### smPPARγOE increased aortic vascular smooth muscle PPARγ expression and activity

**Fig 1:**
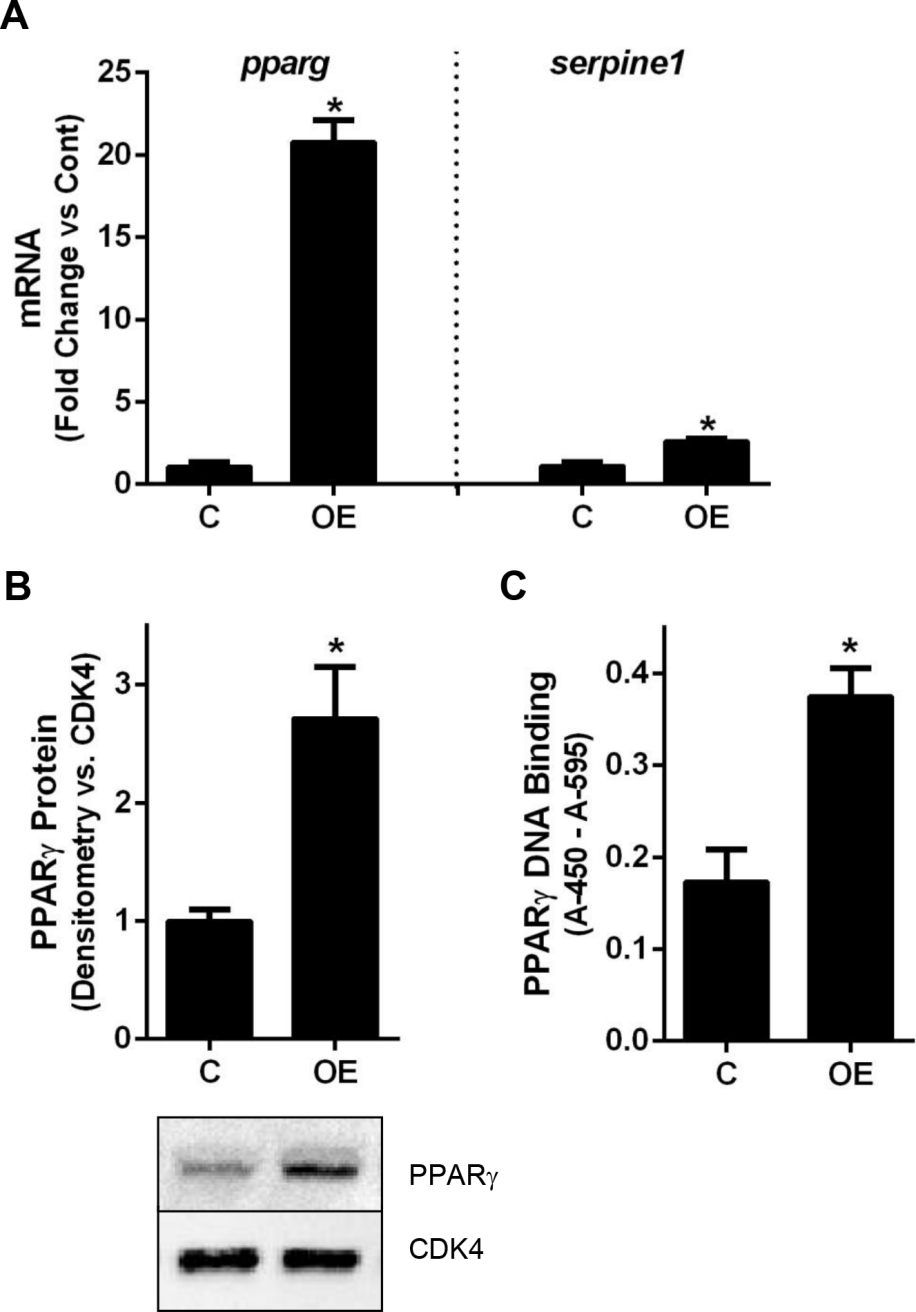
smPPARγOE increased aortic PPARγ expression and activation. **(A)** *pparg* and *serpine1* mRNA levels were measured in smPPARγOE aortas with real-time PCR. Each bar represents the mean ± SEM copies of *pparg* or *serpine1* mRNA normalized to ribosomal protein s9 (*rps9*) in the same sample and expressed as fold change. n = 6. Mice 4–5 weeks post-tamoxifen. **(B)** PPARG protein levels were analyzed with western blotting and densitometry. Each bar represents the mean ± SEM PPARG densitometric intensity relative to cyclin-dependent kinase 4 (CDK4). Representative blots for PPARγ and CDK4 are presented below the graph. n = 5. Mice 3–11 weeks post-tamoxifen. **(C)** PPARγ DNA-binding activity in aortic nuclear extracts was assayed using a TransAM PPARγ ELISA Kit. Each bar represents the mean ± SEM colorometric intensity (absorbance at 450 nm–absorbance at 655 nm). n = 3. Mice 14 weeks post-tamoxifen. *p<0.05 by unpaired t-test.

Six-week old male mice carrying a Cre-controlled constitutively-active PPARγ transgene and a tamoxifen-inducible smooth muscle myosin heavy chain-Cre transgene [23] were injected with tamoxifen to induce Cre recombination and enable smooth-muscle specific transcription of constitutively active PPARγ. To confirm PPARγ overexpression in the vascular smooth muscle layer of smPPARγOE mice, aortas were collected from littermate control and transgenic mice and mRNA, protein, or nuclear proteins were isolated. As illustrated in Fig 1, aortas from smPPARγOE mice displayed a 20-fold increase in *pparg* transcript levels, a 2.5-fold increase in PPARG protein, and a 2-fold increase in PPARγ DNA binding activity. Transcript levels of the PPARγ target gene, *serpine1*, were increased 2-fold in aortas from smPPARγOE mice (Fig 1A), confirming the functional activity of the VP16-PPARγ transgene.

### Characterization of smPPARγOE phenotype

smPPARγOE mice were grossly normal in appearance and behavior. While all mice demonstrated comparable body weights prior to tamoxifen administration, smPPARγOE mice weighed approximately 15% less than littermate control mice by 13 weeks of age (7 weeks following tamoxifen administration; S1 Fig), yet had comparable tibial length (S2 Table). Compared to littermate control mice, smPPARγOE reduced the mass of the perirenal and epididymal fat pads, heart, and kidney, but increased the mass of the testicles, urinary bladder, and gall bladder (S2 Table). Despite reductions in body weight, smPPARγOE mice, compared to littermate control mice, consumed more food and had lower metabolic rates with comparable fasting serum glucose values and rates of insulin-induced glucose disposal (S1 Fig). smPPARγOE mice also displayed polydipsia with reduced urine osmolality and reduced urine concentrations of Na^+^, K^+^, and Cl^-^ (S2 Fig). Serum osmolality (S2 Fig) and hematocrit (not shown) values did not differ between littermate control and smPPARγOE mice.

### smPPARγOE decreased mean arterial pressure and vascular contractility

**Fig 2:**
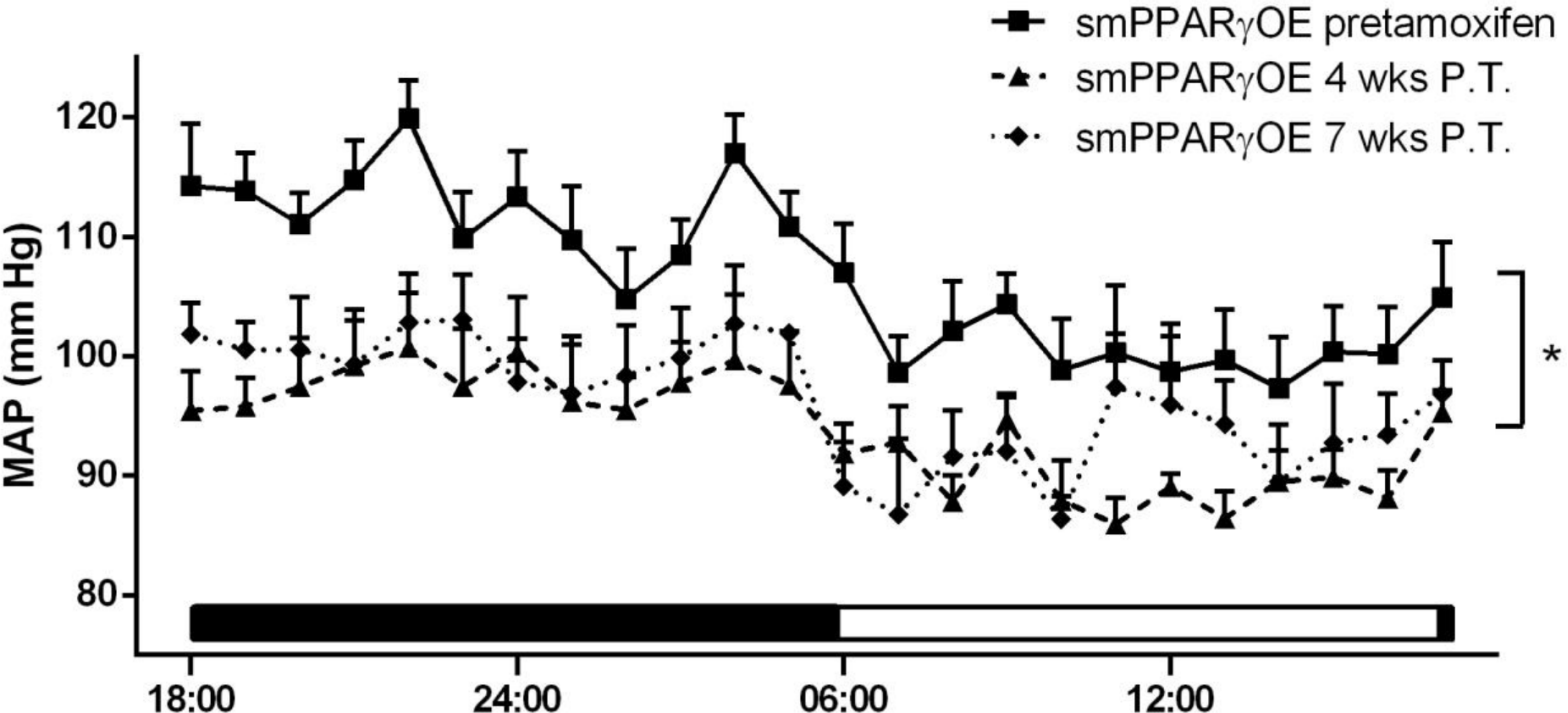
smPPARγOE mice were hypotensive. Telemetric blood pressure transmitters were surgically implanted into mice. Hemodynamic data were collected for 2 days, and mean arterial pressures (MAPs) are presented. Data were collected from mice before tamoxifen and at 4- and 7-weeks post tamoxifen. Each point represents mean MAP ± SEM in mm Hg from 5–7 mice. The bar along the x-axis depicts the light:dark cycle. *p<0.05 vs. smPPARγOE pre-tamoxifen using two-way ANOVA (with repeated measures).

**Fig 3:**
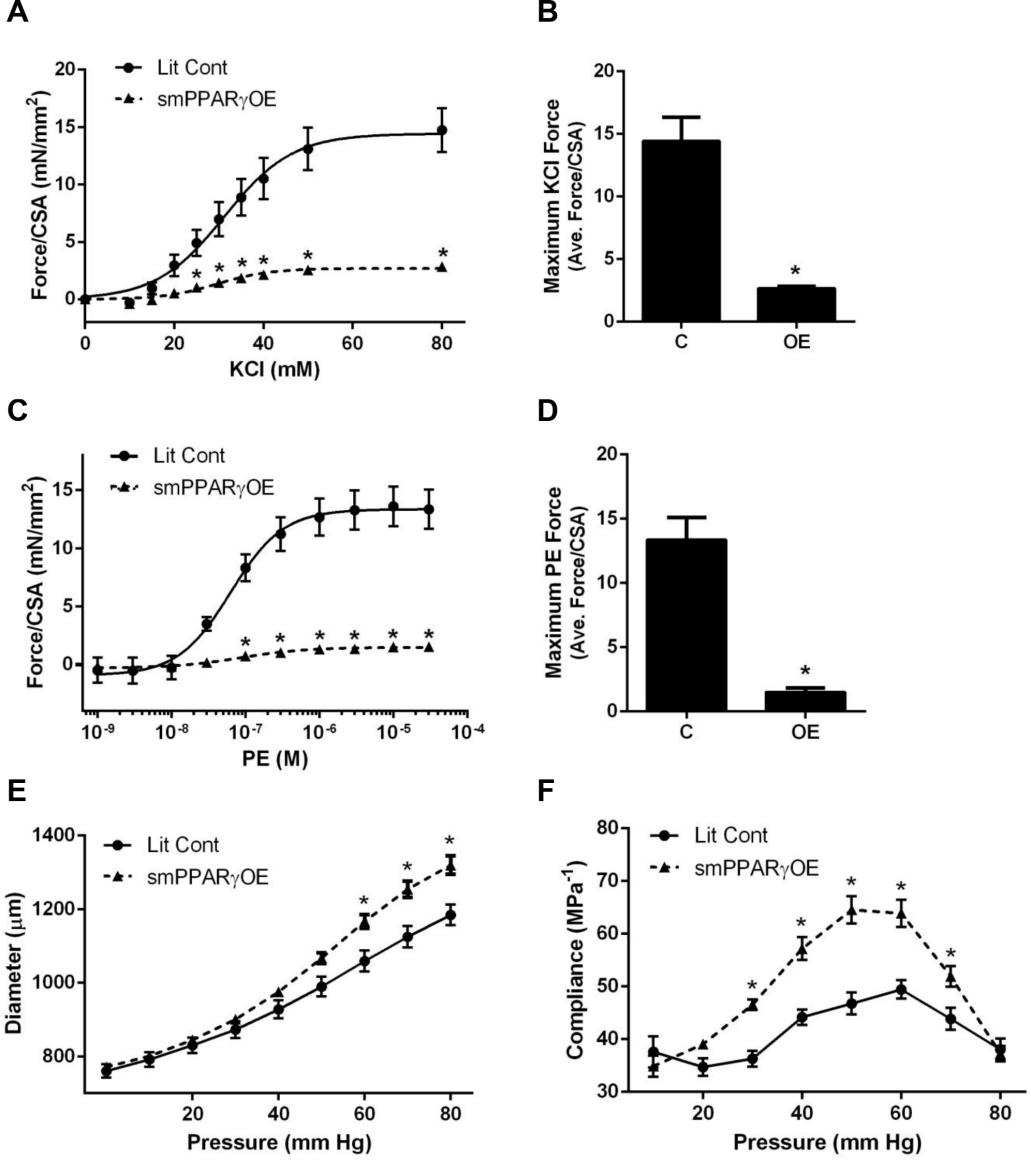
smPPARγOE impaired aortic contractility and enhanced compliance. Freshly dissected aortic rings from littermate control (C) and smPPARγOE (OE) mice were treated with increasing concentrations of KCl in **(A)** or L-phenylephrine (PE) in **(C)**. Each point represents the mean ± SEM force per cross sectional area (CSA, in mN/mm^2^) for 6 animals at 8–14 weeks post-tamoxifen. Cross sectional areas were not different between the two genotypes (Lit Cont = 1.31 ± 0.06 mm^2^; smPPARγOE = 1.24 ± 0.05 mm^2^). Maximal force generation is calculated in **(B)** for KCl and in **(D)** for PE. To determine aortic compliance, vessel segments were incrementally inflated with cell culture medium, and diameters were measured under increasing pressure loads. The mean pressure:diameter measured with *in vitro* stretch for 8 mice at 10 weeks post-tamoxifen is expressed in **(E)**. The calculated compliance (in milli-Pascal^-1^) is indicated in **(F)**. *p<0.05. Panels A, C, E, and F were analyzed by two-way ANOVA with Sidak’s post-test, and Panels B and D were analyzed by unpaired t-test.

Compared to mean arterial pressures before tamoxifen injection, smPPARγOE mice exhibited significant reductions in mean arterial pressure both 4- and 7-weeks post-tamoxifen (Fig 2). Mean arterial pressure in smPPARγOE mice before tamoxifen injection was not different than that recorded in age-matched littermate control animals (data not shown). Heart rates were not different in transgenic mice (7 weeks following tamoxifen injection: littermate control 498.9 ± 34.0 and smPPARγOE 527.0 ± 23.6 beats per minute, 24-hour average, n = 4–5 mice). Heart mass was reduced ~7% in smPPARγOE mice (S2 Table), but differences in wall thickness or ventricular area (S3 Table) were not detected by echocardiography, perhaps due to reduced sensitivity of echocardiography for detecting small changes in cardiac mass. Despite the decreased heart mass in smPPARγOE mice, there were no differences in cardiac output, fractional shortening, ejection fraction, or stroke volume (S3 Table), suggesting that the observed reductions in mean arterial pressures are more attributable to alterations in the systemic vasculature rather than to cardiac dysfunction. To further examine if PPARγ overexpression impacts vascular function, aorta contractile responses were examined using an aortic ring preparation. Isometric forces were measured following cumulative doses of potassium chloride (KCl, Fig 3A and 3B) and phenylephrine (PE, Fig 3C and 3D). Aortas from smPPARγOE mice displayed significant reductions in contractile force per cross sectional area in response to both vasoconstrictors. Maximal contractile force (per cross sectional area) was diminished by 80% in response to KCl (Fig 3B), and almost 90% in response to PE (Fig 3D). In separate studies, pressure:diameter curves were generated by mounting aortas onto glass cannulae and inflating them incrementally with cell culture medium. The resulting aortic diameters were measured and graphed relative to corresponding pressure loads (Fig 3E). Compared to littermate controls, higher levels of deformation were observed at pressures ≥ 50 mm Hg in smPPARγOE aortas. Similarly, stiffness calculations revealed significant increases in aortic compliance in smPPARγOE mice between 30–70 mm Hg (Fig 3F).

### smPPARγOE decreases expression of contractile markers and myocardin in aortic and mesenteric vessels

**Fig 4:**
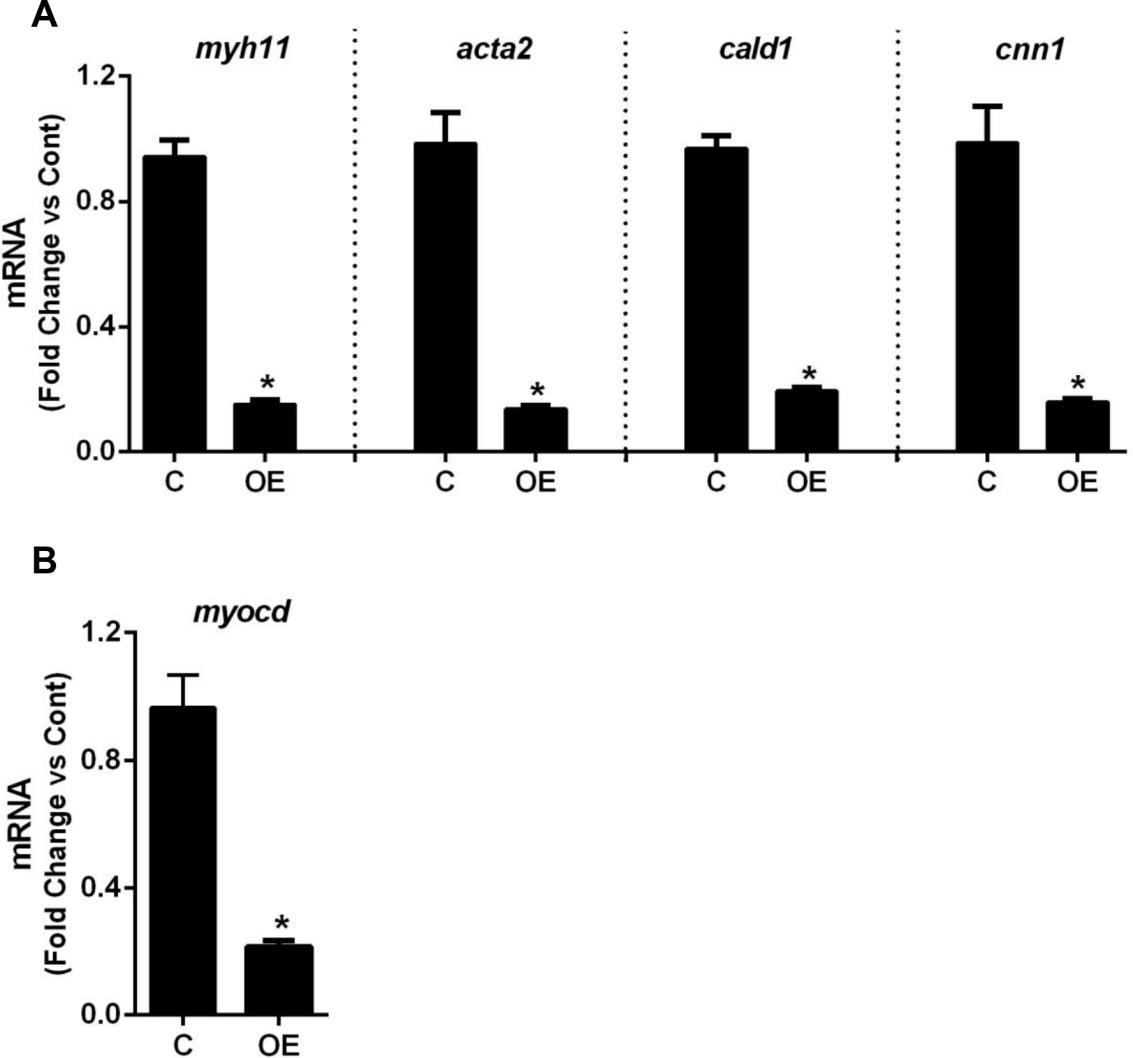
smPPARγOE decreased the transcripts of aortic smooth muscle contractile proteins and the smooth muscle transcriptional co-activator, myocardin. mRNA levels of smooth muscle myosin heavy chain (*myh11*), alpha smooth muscle actin (*acta2*), caldesmon (*cald1*), and calponin (*cnn1*) were measured in aortas with qRT-PCR **(A)**. mRNA levels of myocardin (*myocd*) are shown in **(B)**. Each bar represents the mean ± SEM copies of each transcript normalized to ribosomal protein 9s (*rps9*) in the same sample and expressed as fold change vs control. n = 6. Mice 4–5 weeks post-tamoxifen. *p<0.05 using unpaired t-test.

To further examine the mechanisms underlying altered vascular contractile responses in smPPARγOE mice, the levels of several vascular contractile proteins were examined. qRT-PCR analysis demonstrated significant reductions in transcripts for the smooth muscle contractile proteins, smooth muscle myosin heavy chain (*myh11*), α-smooth muscle actin (*acta2*), caldesmon (*cald1*), and calponin (*cnn1*, Fig 4A). These transcripts were also reduced (>50%) in the mesenteric artery (panel C in S3 Fig), indicating that these derangements exist within resistance vessels as well. Corresponding reductions in aortic protein levels of ACTA2, CALD1, and CNN1 were also observed (S4 Fig). These contractile genes contain serum response factor (SRF) binding sites within their promoters. Since SRF binding is crucially dependent on the presence of myocardin [33], we examined the levels of this co-activator in the transgenic vasculature and found that *myocd* transcripts were reduced~80% in transgenic aortas (Fig 4B) and ~60% in transgenic mesenteric arteries (panel C in S3 Fig).

### smPPARγOE causes aortic dilatation and derangements in the architecture of the vascular wall

**Fig 5:**
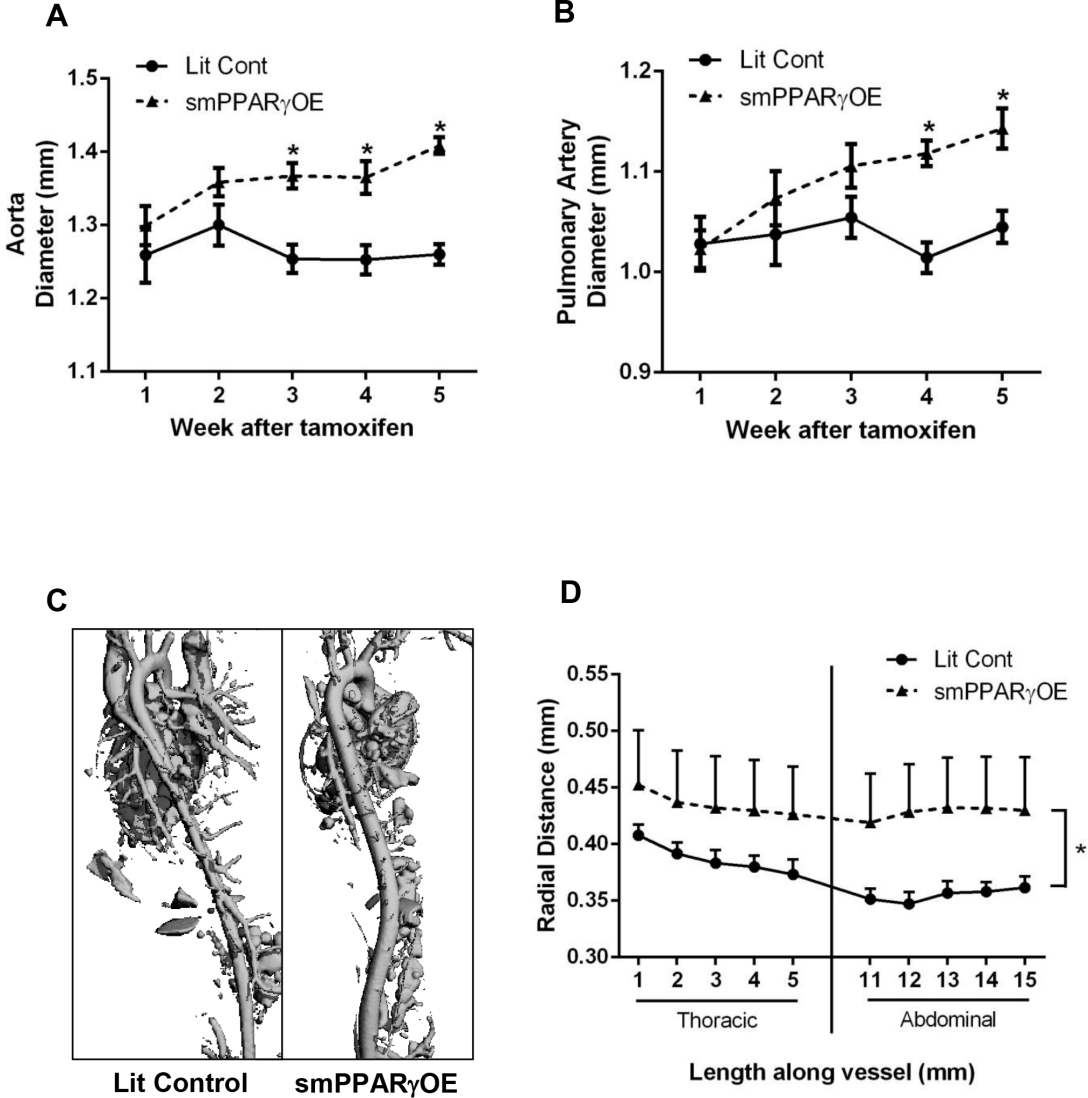
smPPARγOE mice developed aortic dilatation. Echocardiography was performed weekly before and after tamoxifen injection (up to 5 weeks post-tamoxifen), and measurements of aortic **(A)** and pulmonary artery **(B)** diameters were performed. Each point represents the mean ± SEM diameter in mm. n = 7. *p<0.05 using 2-way ANOVA with repeated measures and Sidak’s post-test. The longitudinal extent of the aortic dilatation was further assessed with micro-CT. Representative images of micro-CT angiographs are displayed in the posterior-to-anterior projection in **(C)**. Serial digital measurements of the thoracic and abdominal aorta were analyzed from 3–4 littermate control and smPPARγOE mice at 10 weeks post-tamoxifen. The mean aortic radius was graphed according to distance from the arch **(D)**. *p<0.05 using 2-way ANOVA (with repeated measures).

Initial echocardiography revealed that smPPARγOE mice had increased aortic and pulmonary artery diameters relative to littermate control mice. To further explore the temporal onset of aortic dilatation *in vivo*, serial echocardiograms were performed after the administration of tamoxifen, and aortic and pulmonary artery diameters were measured. Whereas, proximal aortic and pulmonary artery diameters were similar between littermate control and smPPARγOE aortas 1–2 weeks after tamoxifen administration, 3-weeks following tamoxifen treatment, aortic diameters from smPPARγOE mice were significantly larger than littermate control aortas (Fig 5A) and the diameter of smPPARγOE pulmonary arteries began increasing 4-weeks post-tamoxifen (Fig 5B). To examine aortic dilatation in more distal segments, mice were imaged by micro-CT 7-weeks after tamoxifen injection (Fig 5C). Measurements of the mean aortic diameter from the thoracic and abdominal aortas demonstrated significant enlargement of the smPPARγOE aortas (Fig 5D). The absolute values for aortic diameters measured with echocardiography or CT differ because they are performed on different regions of the aorta (above or below the aortic arch, respectively) and the state of the vessel (*in vivo* unfixed or *ex vivo* fixed, respectively). Regardless of measurement technique, Fig 5 emphasizes that smPPARγOE causes aortic dilatation.

**Fig 6:**
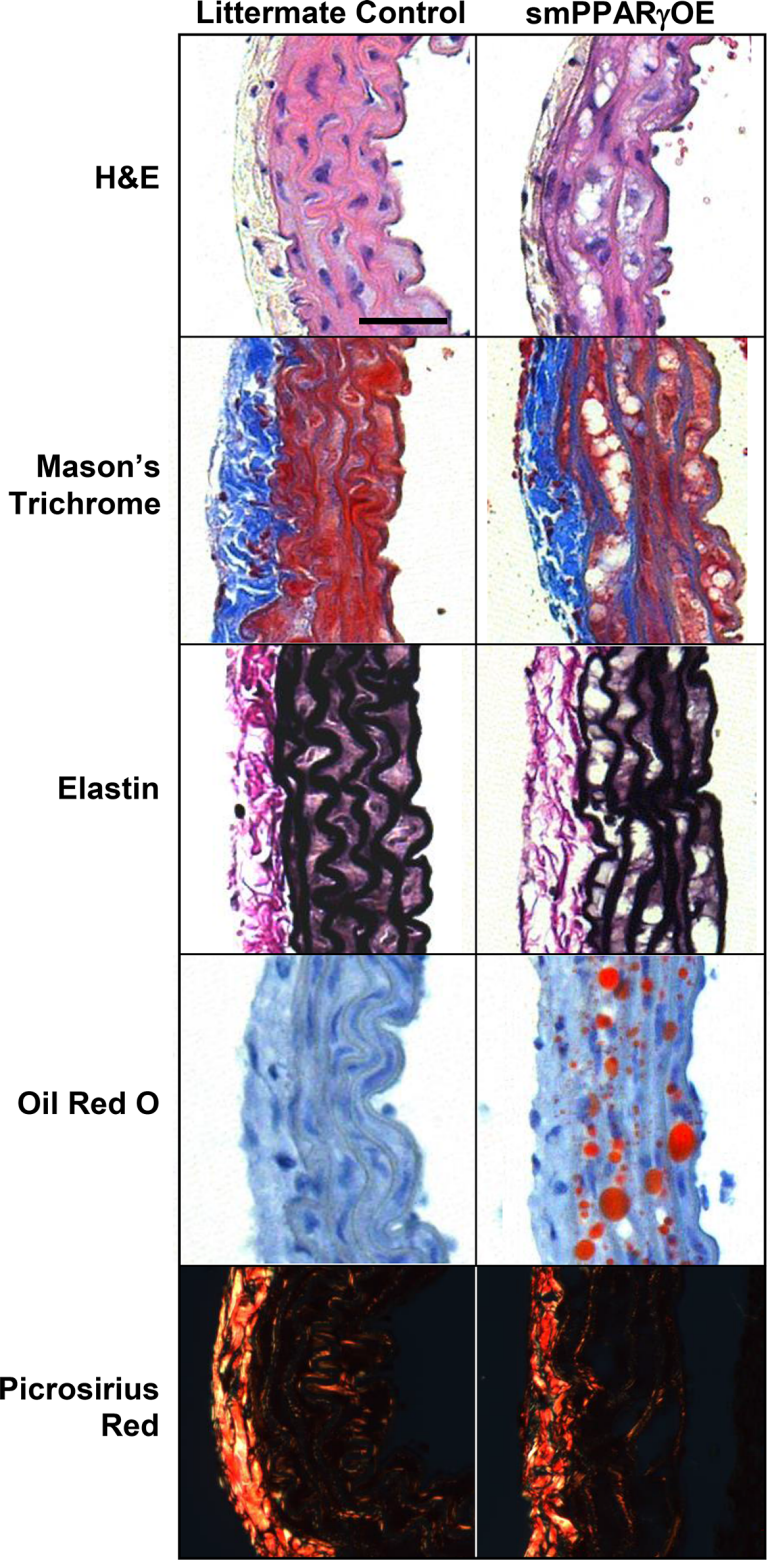
smPPARγOE altered the architecture of the vascular wall. Four-weeks following tamoxifen injection, the descending aortas from littermate control and smPPARγOE mice were isolated, fixed, and embedded in paraffin (except oil red O stain, which utilized frozen tissue blocks). Cross sections were stained with H&E, Mason’s trichrome, elastin stain, oil red O, or picrosirius red. Sections were examined at 20x with light microscopy or polarized light microscopy (picrosirius red). Representative images are presented. Scale bar = 50 μm.

**Fig 7:**
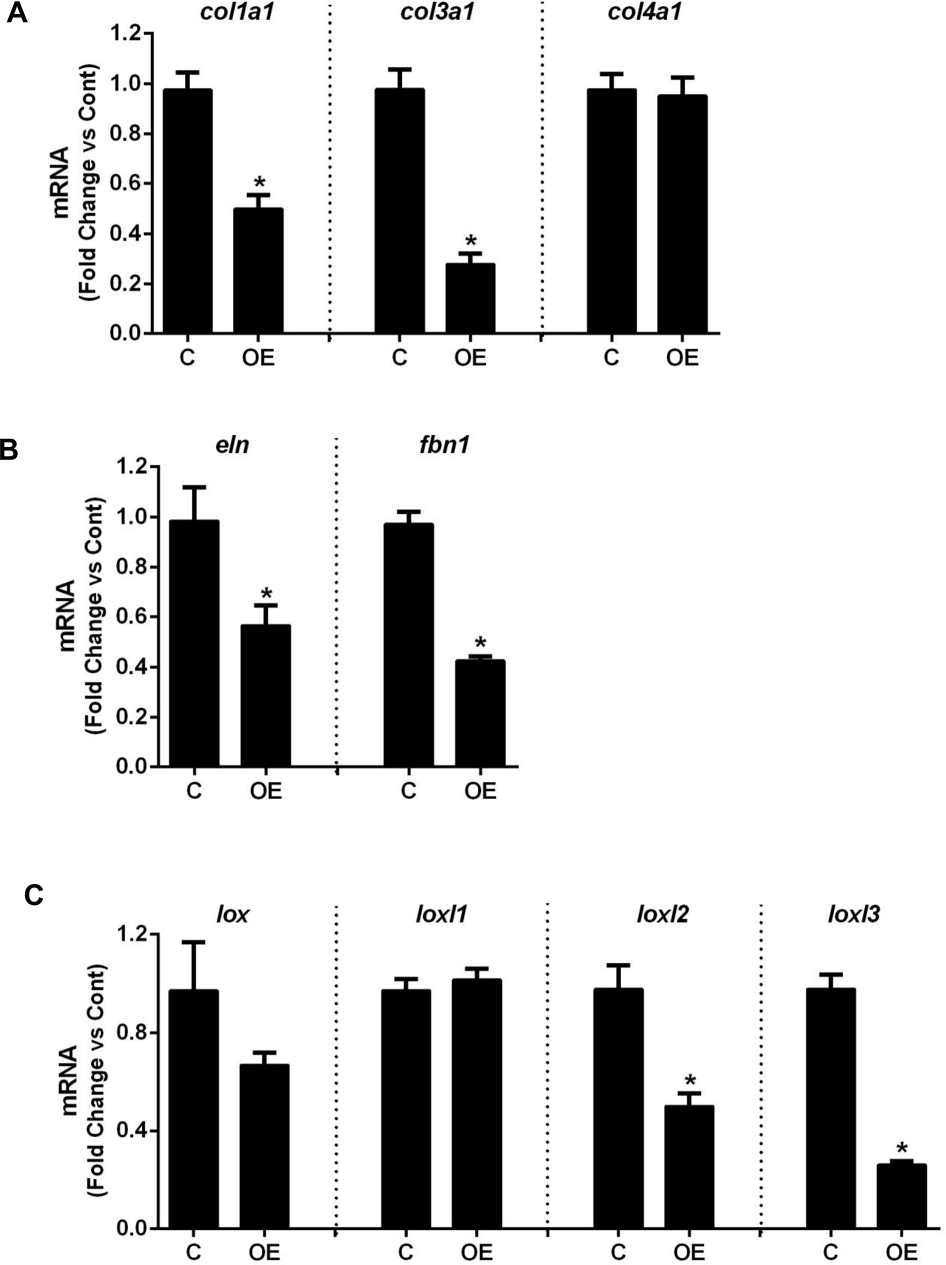
smPPARγOE altered mRNA levels of matrix molecules. mRNA levels for collagen **(A)**, elastin and fibrillin-1 **(B)**, and the lysyl oxidase family members **(C)** were measured in aortas from littermate control (C) or smPPARγOE (OE) mice by qRT-PCR. Each bar represents the mean ± SEM copies of each mRNA normalized to *rps9* in the same sample and expressed as fold change vs C. n = 6. Mice were 4–5 weeks post-tamoxifen. *p<0.05 using unpaired t-test.

To further examine the mechanisms for aortic dilatation, histological analysis was performed. Defects in the aortic media were observed in sections from smPPARγOE mice stained with H&E, mason’s trichrome, or elastin stains (Fig 6). Oil red O staining revealed that these defects were filled with lipid. Similar lipid deposition was observed in resistance vessels (mesenteric artery, panel A in S3 Fig) as well as other smooth muscle-rich tissues from smPPARγOE mice including bladder and intestine. The former was associated with gross dilatation of the urinary and gall bladders (S5 Fig). Also noted were differences in the morphology of the elastic laminae in aortas from smPPARγOE mice and the suggestion of reduced collagen deposition in the medial layer of sections from transgenic mice stained with picrosirius red. These alterations appeared to progress over time following tamoxifen-induced recombination, as suggested by the more pronounced histological derangements in transgenic mice 28 weeks after tamoxifen injection (S6 Fig). To further explore underlying causes for the structural alterations in the aortic wall, levels of aortic connective tissue components were quantitatively assessed. qRT-PCR demonstrated reductions in mRNA levels of collagen type I, alpha 1 (*col1a1*, reduced ~40%) and collagen type 3, alpha 1 (*col3a1*, reduced ~60%), whereas mRNA levels of collagen type 4, alpha 1 (*col4a1*) were unchanged (Fig 7A). At the same time, aortic mRNA levels of elastin (*eln*) and the elastic fiber component fibrillin-1 (*fbn1*) were reduced 40% and 60%, respectively, in smPPARγOE mice (Fig 7B). Similarly, the mRNA levels for enzymes involved in elastin cross-linking were selectively decreased in smPPARγOE aortas. For example, lysyl oxidase-like protein 2 (*loxl2*) and *loxl3* transcripts were reduced in smPPARγOE aortas whereas *lox* and *loxl1* transcripts were unchanged (Fig 7C).

### smPPARγ overexpression stimulates expression of adipocyte markers in the vascular wall *in vivo* and in smooth muscle cells *in vitro*

**Fig 8:**
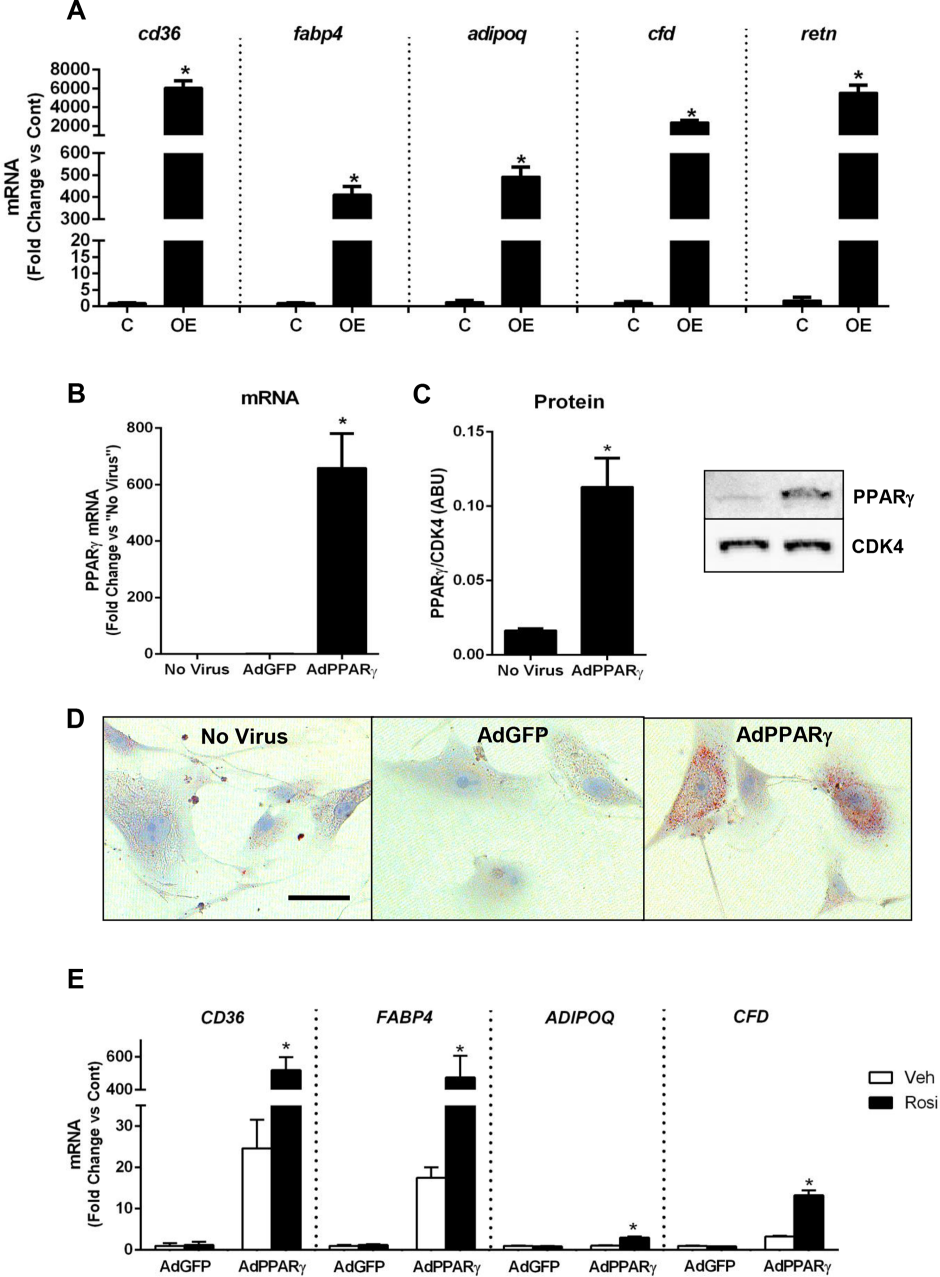
smPPARγOE increased mRNA levels of adipocyte-related genes *in vivo* and *in vitro*. RNA was isolated from aortas of littermate control (C) or smPPARγOE (OE) mice and subjected to qRT-PCR for selected adipocyte-related genes. In **(A)** levels of fatty acid translocase (*cd36*), fatty acid binding protein 4 (*fabp4*), and other adipocyte markers are presented for mice 4–5 weeks post-tamoxifen. Each bar represents the mean ± SEM copies of each mRNA normalized to *rps9* in the same sample and expressed as fold change vs control. n = 6. *p<0.05 using unpaired t-test. In **(B-E)**, adenovirus was used to upregulate PPARγ *in vitro* using hAoSMCs. Cells were treated with no virus, Ad-GFP, or Ad-PPARγ. After 5 hours, the media were replaced with growth media. 96 hours following infection, mRNA and protein were collected. PPARγ mRNA levels **(B)** and protein levels **(C)** are displayed. In **(D)**, hAoSMCs were plated onto chamber slides and treated with adenoviruses. 7-days after infection, the cells were fixed and stained with oil red O (red staining indicates lipid). Representative images are shown at 20x. Scale bar = 50 μm. In **(E)**, hAoSMCs were infected with Ad-GFP or Ad-PPARγ for 5 hours. To stimulate PPARγ activity, selected cells were treated with rosiglitazone (10 μM x 72 hours). 96 hours following infection, RNA was isolated and qRT-PCR performed. mRNA expression levels of adipocyte-related genes were determined. Each bar represents the mean ± SEM copies of each mRNA normalized to *RPS9* in the same sample and expressed as fold change vs control. n = 3–6. *p<0.05 vs all groups using two-way ANOVA with Sidak’s post-test.

PPARγ transactivates a program of gene expression that stimulates adiopocyte differentiation. In the vascular wall of smPPARγOE mice, the expression of several adipocyte, PPARγ-controlled genes was dramatically increased including fatty acid translocase (*cd36*), fatty acid binding protein 4 (*fabp4*), and the adipose-derived hormones, adiponectin (*adipoq*), adipsin (*cfd*), and resistin (*retn*, Fig 8A). Transcripts of these adipocyte-related genes were also upregulated in resistance vessels (mesenteric artery, panel D in S3 Fig).

Two complementary approaches were used to determine if PPARγ overexpression is sufficient to induce an adipocyte phenotype in smooth muscle cells *in vitro*. Human aortic smooth muscle cells (hAoSMCs, Lonza) were infected with PPARγ adenovirus which effectively increased PPARγ mRNA and protein levels relative to controls (Fig 8B and 8C). Similar to the smooth muscle cells of smPPARγOE mice, cultured hAoSMCs treated with AdPPARγ also accumulated lipids, as visualized by oil red O staining. To further assess PPARγ-regulated gene expression in hAoSMCs, mRNA was isolated from hAoSMCs transfected with adenovirus (AdPPARγ or AdGFP) ± rosiglitazone treatment (to stimulate PPARγ activity). Similar to transgenic mouse tissues, mRNA levels of adipocyte-related genes were significantly elevated in hAoSMC treated with AdPPARγ + rosiglitazone including *CD36*, *FABP4*, *ADIPOQ*, and *CFD* (Fig 8E). However, unlike the smPPARγOE aortas, AdPPARγ delivery did not alter transcript levels of *ACTA2*, *CALD1*, or *CNN1* (data not shown), potentially because smooth muscle cells rapidly lose the contractile phenotype and contractile protein expression in culture even in the absence of PPARγ stimulation [34]. Similarly, mouse aortic smooth muscle cells were isolated as reported [35] and treated with or without tamoxifen *in vitro*. Tamoxifen-induced recombination and PPARγ overexpression *in vitro* stimulated increased lipid uptake that was absent in mouse aortic smooth muscle cells not treated with tamoxifen (S8 Fig).

## Discussion

**Fig 9:**
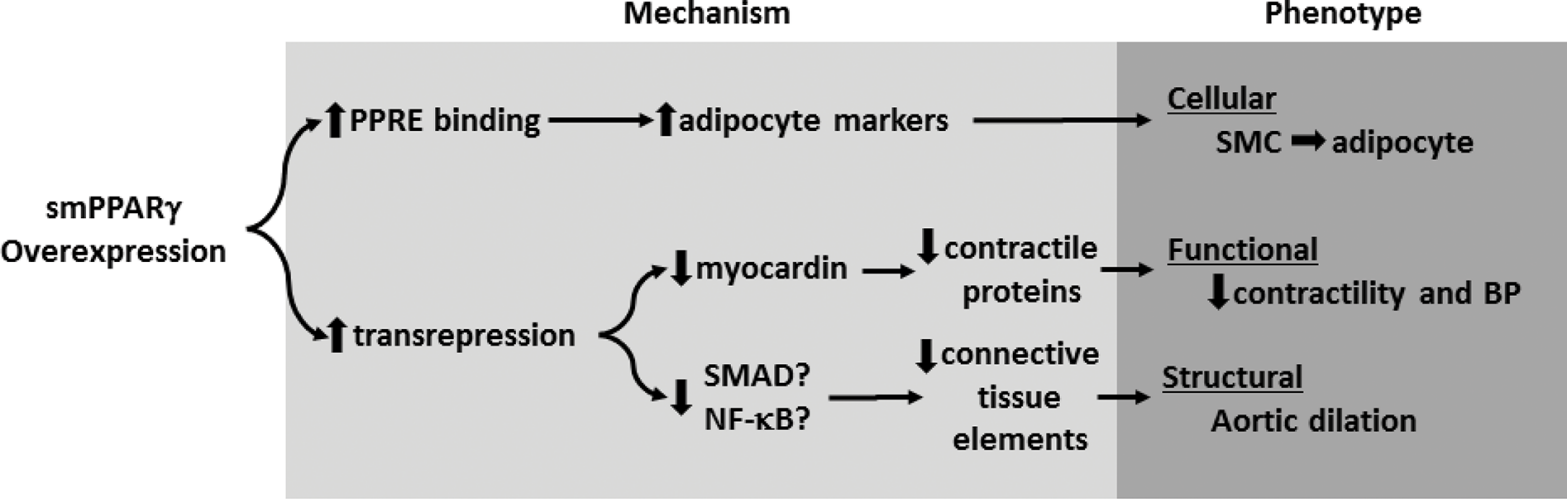
Schema of putative mechanisms underlying the altered vascular phenotype of smPPARγOE mice. Enhanced expression of smooth muscle cell PPARγ increases the expression of PPRE-containing adipocyte genes while simultaneously reducing the expression of contractile proteins and connective tissue components through transrepression. Collectively, these alterations in gene expression stimulate smooth muscle cell to adipocyte transdifferentiation and the observed alterations in vascular structure and function.

To mitigate the limitations of approaches employing PPARγ ligands to study the role of PPARγ gain-of-function in vascular pathophysiology, we employed a smooth muscle-targeted PPARγ overexpressing mouse model. To our knowledge, this report is the first to comprehensively examine the vascular phenotype of smooth muscle cell-targeted PPARγ overexpression *in vivo*. The current results (summarized in Fig 9) demonstrate that smooth muscle-targeted PPARγ overexpression exerts profound effects on vascular structure and function. smPPARγOE caused 2–3 fold increases in PPARγ protein, DNA binding activity, and target gene expression. Although smooth muscle-targeted PPARγOE had no significant effect on echocardiographic parameters of left ventricular function, it lowered mean arterial blood pressure suggesting a decrease in vascular resistance. Additionally, aortic rings from transgenic vessels had dramatically reduced contractile responses to both KCl and PE. These impairments in contractile response appear to be the direct result of decreased contractile proteins in the vascular wall of conduit and resistance vessels, including *myh11*, *acta2*, *cald1*, and *cnn1*. Reductions in these contractile proteins were associated with and likely attributable to reductions in myocardin, a co-activator of the smooth-muscle transcription factor, SRF. Consistent with this hypothesis, myocardin expression was reduced in smPPARγOE aorta and mesenteric artery. While it seems reasonable to postulate that the reduction in myocardin contributes to the decrease in contractile protein expression, PPARγ-induced myocardin inhibition, to our knowledge, has not been previously described. However, since PPARγ activation inhibits NFAT (unpublished data) and NFATc3 directs the expression of myocardin [36], it is tempting to speculate that PPARγactivation causes transrepression of transcription factors that stimulate myocardin expression [37]. Alternatively, miR-9 inhibits the expression of myocardin, and *in silico* analysis suggests that the pri-mir-9 promoter region contains five putative PPAR response elements [38]. These considerations indicate that PPARγ activation could enhance post-transcriptional pathways that downregulate myocardin expression. Further studies will be required to test these hypothesis.

We also postulate that the reductions in collagen and elastic fiber components in the medial layer of smPPARγOE aortas contributed to the observed increases in vascular compliance and dilatation. Since many collagen [39] and lysyl oxidase family members [40] are regulated by TGFβ in vascular smooth muscle cells, we postulate that PPARγ-induced transrepression of Smad and NF-κB activation may contribute to reductions in the expression of these vascular wall components [41].

Not only did smPPARγOE cause loss of aortic collagen and elastin components, it also stimulated the expression of PPRE-containing genes including the adipocyte lineage markers *cd36*, *fabp4*, *cfd*, and *retn*, leading to an accumulation of lipid aggregates in the vascular wall as detected by oil red O staining. Comparable alterations were observed *in vitro* in hAoSMC treated with PPARγ adenovirus, suggesting that smPPARγOE is sufficient to induce vascular smooth muscle to adipocyte transdifferentiation. These *in vivo* findings extend previous evidence that adenoviral-PPARγ overexpression in rat vascular smooth muscle cells *in vitro* stimulated increased *cd36* expression, enhanced lipid uptake, and loss of contractile protein expression [42]. Collectively, these findings present a novel example of vascular smooth muscle cell phenotypic switching not previously observed *in vivo* that includes the activation of adipocyte-related genes in resident vascular smooth muscle cells. These findings provide additional support for the remarkable phenotypic plasticity of vascular smooth muscle cells *in vivo* [43].

smPPARγOE not only caused a profound vascular phenotype but also induced several other derangements less well characterized in our study. smPPARγOE caused significant weight loss without affecting growth (tibial length), small but significant increases in food consumption and reductions in metabolic rate, and no alteration in basal glucose levels or insulin-induced glucose disposal. Increased lipid uptake was also observed in the smooth muscle layers of other structures in smPPARγOE mice including the urinary and gall bladders. Because these bladders were grossly distended at autopsy, based on the contractile dysfunction in the vasculature, it is tempting to speculate that smPPARγOE causes diffuse smooth muscle contractile dysfunction. Impaired contractility in the gastrointestinal tract might therefore contribute to impaired gut motility, altered nutrient absorption, and weight loss. In addition, smPPARγOE caused significant reductions in the mass of visceral fat pads. This confirms previous reports that manipulation of smooth muscle PPARγ function can impact extra-smooth muscle cell adipose tissue and fat metabolism [44]. Finally, the polydipsia and reduced urine osmolality observed in smPPARγOE mice may indicate impaired urinary concentrating ability due to altered medullary blood flow. Additional studies will be required to further characterize these alterations in renal function in smPPARγOE mice.

The findings in this model have potential implications for clinical disorders of the aorta.

Cystic medial degeneration is the histopathological process seen in the aortas of patients with Marfan syndrome, congenital aortic disease, atherosclerosis, and aging who have annuloaortic ectasia. In these conditions, aortic dilatation is characterized by disruption of elastic elements, loss of vascular smooth muscle cells, and accumulation of proteoglycans in the vascular media. Previous analysis demonstrated that PPARγ expression was increased in aneurysms of Marfan aortas with pathological cystic medial degeneration, and the severity of cystic medial degeneration and aortic diameter were positively correlated with the degree of PPARγ expression suggesting that increased expression of vascular smooth muscle PPARγ may contribute to the pathogenesis and progression of cystic medial degeneration [45]. The current study provides the first description that smPPARγOE is sufficient to induce structural changes in the aortic wall that cause dilatation. The molecular mechanisms for these alterations in vascular structure include smooth muscle cell to adipocyte transdifferentiation and significant alterations in the expression of connective tissue elements. For example, because Marfan Syndrome is caused by mutation of the fibrillin-1 gene, it is tempting to speculate that smPPARγOE-induced reductions in fibrillin-1 contribute to the structural alterations in the vascular wall.

Little information is available regarding the impact of increased PPARγ expression on vascular endpoints. In a balloon injury model of vascular remodeling, adenoviral transfection of PPARγ attenuated, whereas dominant negative PPARγ exacerbated, vascular neointimal formation and vascular cell proliferation *in vivo* [46]. While adipoctye-targeted PPARγ overexpression exerted significant effects on metabolism and caused degrees of insulin sensitization that were comparable to those caused by pioglitazone therapy [47], our results suggest that robust overexpression of PPARγ in vascular smooth muscle cells fails to alter glucose disposal but induces smooth muscle cell to adipocyte transdifferentiation sufficient to cause structural and functional vascular derangements *in vivo*. However, the pathobiological relevance of this degree of transgene overexpression is more difficult to assess. Taken together with previous reports demonstrating vascular dysfunction following loss of smooth muscle cell PPARγ activity, the current study suggests that normal vascular function is associated with optimal levels of PPARγ activity. The relationship between vascular dysfunction and vascular cell PPARγ appears best described by a “U” relationship (S9 Fig). At the extremes, absence of PPARγ expression or its robust overexpression are sufficient to induce significant functional and perhaps structural derangements in the vasculature. At more intermediate levels of PPARγ activity, pharmacological tuning to increase or decrease PPARγ activity in pathophysiological states may provide opportunities for therapeutic targeting. Future studies examining the impact of PPARγ activity on vascular function in health and disease will need to define not only the degree of activation / deactivation of this receptor but also by the cellular locale in which these alterations occur, the specific ligands or antagonists employed to alter PPARγ and the conformational changes they induce in the PPARγ receptor that recruit or de-recruit unique combinations of coactivators and corepressors to the transcriptional complex.

## Supporting Information

S1 Table Primer sequences used for quantitative RT-PCR. Mouse primers in lowercase. Human primers in uppercase.
(TIF)

S2 Table Tissue weights and tibial length in littermate control and smPPARγOE mice. Selected organs and tissues were harvested from 11 week-old mice (5 weeks after tamoxifen injection) and weighed or measured. Organ and fat pad measurements are presented as organ weight (mg) relative to total body weight (g) ± SEM. Kidney and testicle data are presented as pair average (mg) / total body weight (g). *p<0.05 by unpaired t-test.
(TIF)

S3 Table Cardiac function assessed by echocardiography in smPPARγOE and littermate control mice. Echocardiographic data from littermate control (Lit Cont) and smPPARγOE mice at 4 weeks post-tamoxifen. Values represent mean ± SEM from 7 animals. Unpaired t-tests revealed no significant differences between Lit Cont and smPPARγOE mice.
(TIF)

S1 Fig smPPARγ overexpression inhibited weight gain, increases food consumption, and decreases metabolic rate. 6 week old littermate control (Lit Cont or C) and smPPARγOE (OE) mice were injected with tamoxifen (50 mg/kg/day for 5 days). Body weights of 13 mice were tracked over time **(A)**. Data points represent mean ± SEM body mass in grams. *p<0.05 using 2-way ANOVA with repeated measures and Sidak’s post-test. Mice (14 weeks after tamoxifen-induced recombination) were housed singly, and food consumption was observed over 24 hours **(B)**. Each bar represents the mean ± SEM food consumed in grams from 5–6 animals. *p<0.05 using unpaired t-test. Mice were then placed in an Oxymax Lab Animal Monitoring System to determine the volume of O_2_ consumed and CO_2_ produced. Volumes were measured for 60 seconds and sampled every 30 minutes for 24 hours. Energy expenditure (heat) was calculated as CV*VO_2_, where CV = 3.815+[1.232*(VCO_2_/VO_2_)]. The data over 24 hours were averaged and graphed in **(C)**. Each bar represents the group mean ± SEM heat (kcal/hr) from ten mice. *p<0.05 using unpaired t-test. In **(D)**, intraperitoneal glucose injections were administered to Lit Cont and smPPARγOE mice four week post-tamoxifen, and serum glucose values were determined with an Accu-check Aviva glucose meter at intervals for 4 hours. Data points represent the mean ± SEM blood glucose in mg/dL from 9 mice.
(TIF)

S2 Fig smPPARγOE mice displayed polydipsia and reduced urine osmolality. In **(A),** 14-weeks following tamoxifen-induced recombination, littermate control (C) and smPPARγOE (OE) mice were housed singly and water consumption was measured for 24 hours. Each bar represents the mean ± SEM water consumed (grams) per mouse from 5–6 mice. After sacrifice, urine and blood samples were collected simultaneously. Urine **(B)** and serum **(C)** osmolality were measured using an osmometer in mice following *ad lib* water intake. Bars represent the mean ± SEM osmolality (mOsmol/kg) from 3–5 animals. In **(D)**, urine Na^+^, K^+^, and Cl^-^ levels were measured and expressed relative to urine creatinine levels. n = 3–4. For all, *p<0.05 using unpaired t-tests.
(TIF)

S3 Fig Mesenteric arteries from smPPARγOE mice display abnormal lipid deposition, reduced mRNA levels of contractile proteins, and increased mRNA levels of adipocyte markers. **(A)** Four to six weeks following tamoxifen injection, the intestinal branches of the superior mesenteric artery were isolated from littermate control and smPPARγOE mice. Vessels were cleaned of adipose tissue and frozen in OCT blocks. Cross sections were stained with an oil red O kit. Sections were examined at 20x with light microscopy. Red = lipid. *Scale bar* = 35 µm. Separately, mesenteric arteries were collected, pooled (two per sample), and RNA isolated. qRT-PCR was performed. *pparg* and downstream target gene *serpine1* are shown in **(B)**. Contractile proteins are displayed in **(C)** and adipocyte-related markers are displayed in **(D)**. Each bar represents the mean ± SEM copies of mRNA normalized to *rps9* in the same sample and expressed as fold change vs C. n = 4. *p<0.05 by unpaired t-tests.
(TIF)

S4 Fig smPPARγOE decreased aortic smooth muscle contractile proteins. Alpha smooth muscle actin (ACTA1), caldesmon (CALD1), and calponin (CNN1) protein levels were determined by western blot and densitometry in whole aortic lysates. Each bar represents the mean ± SEM protein densitometric intensity relative to CDK4 and expressed as fold change vs control for 3–5 animals at 3–11 weeks post-tamoxifen. Representative blots are presented below the graphs. *p<0.05 using unpaired t-test.
(TIF)

S5 Fig Enhanced lipid deposition in smooth muscle layers of smPPARγOE mice. In **(A)**, frozen sections from the bladder, intestine, and heart of littermate control (C or Cont) and smPPARγOE (OE) mice were stained with oil red O. Representative images are shown. Arrow = large artery. *Scale bars* = 100 μm. In **(B)**, representative images of the urinary bladder (top) and gall bladder (bottom) from LC and OE mice are presented. The bladders were then removed, drained, and weighed. Each bar represents the mean ± SEM urinary or gall bladder weight from 5–6 mice, four weeks post-tamoxifen. *p<0.05 using unpaired t-test.
(TIF)

S6 Fig Derangements in vascular wall architecture in smPPARγOE mice worsened over time. 28-weeks following tamoxifen-induced recombination, the descending aortas from littermate control and smPPARγOE mice were isolated, fixed, and embedded in paraffin (except oil red O stain which utilized frozen tissue blocks). Cross sections were stained with H&E, Mason’s trichrome, elastin, oil red O, or picrosirius red stains. The resulting sections were examined at 20x using light microscopy or polarized light microscopy (picrosirius red). Representative images are presented. *Scale bar* = 50 μm.
(TIF)

S7 Fig PPARγ overexpression in smPPARγOE mice is restricted to the smooth muscle-rich medial layer in aortas. 8-weeks following tamoxifen-induced recombination, the descending aortas from littermate control and smPPARγOE mice were embedded in Optimal Cutting Temperature medium and frozen. 5μm cross sections were blocked and incubated with PPARγ (Santa Cruz sc-7273, mouse monoclonal, 1:50) and smooth muscle actin (Thermo Fisher RB-9010-P, rabbit polyclonal, 1:50) antibodies. The PPARγ signal was fluorescently labeled using a mouse-on-mouse fluorescein kit (Vector Laboratories FMK-2201). The smooth muscle actin was visualized with a rhodamine red-labeled anti-rabbit antibody (1:100). The resulting slides were examined under fluorescence at 20x. Representative images are presented from an n = 4. *Scale bar* = 50 μm.
(TIF)

S8 Fig Smooth muscle cells isolated from the aortas of smPPARγOE mice accumulated lipids *in vitro*. Aortas from non-induced smPPARγOE mice were digested with collagenase, and smooth muscle cells were cultured onto chamber slides. Cells were treated with vehicle (0.01% ethanol) or tamoxifen (1 μg/ml) daily for 5 days. On day 14, cells were fixed with 10% formaldehyde and stained with oil red O. Representative images are shown at 40x. *Scale bar* = 50 μm.
(TIF)

S9 Fig Putative relationship between vascular smooth muscle cell (VSMC) PPARγ activity and vascular dysfunction. Current evidence indicates that both low and high levels of PPARγ activity in VSMC perturb vascular homeostasis suggesting that intermediate levels are required for normal vascular function and that therapeutic targeting of vascular PPARγ activity might enable reductions in selected pathophysiological derangements in vascular function caused by altered PPARγ activity.
(TIF)

S1 Raw Data Aortic histology: Elastic stain images. (PPTX)

S2 Raw Data Aortic histology: H&E stain images. (PPTX)

S3 Raw Data Aortic histology: Oil Red O stain images. (PPTX)

S4 Raw Data Aortic Histology: Picrosirius Red stain images. (PPTX)

S5 Raw Data Aortic Histology: Trichrome stain images. (PPTX)

S6 Raw Data MicroCT images. (PPTX)

S7 Raw Data Western blot images. (PPTX)

S8 Raw Data hAoSMC Oil Red O stain images. (PPTX)

S9 Raw Data Numerical data, part 1 (Prism file). (PZF)

S10 Raw Data Numerical data, part 2 (Prism file). (PZF)
