# Supplementary figures and images for: Smooth Muscle-Targeted Overexpression of Peroxisome Proliferator Activated Receptor-γ Disrupts Vascular Wall Structure and Function
Source: PLoS One. 2015 Oct 9;10(10):e0139756. doi: 10.1371/journal.pone.0139756 (PMC4599849; doi:10.1371/journal.pone.0139756)

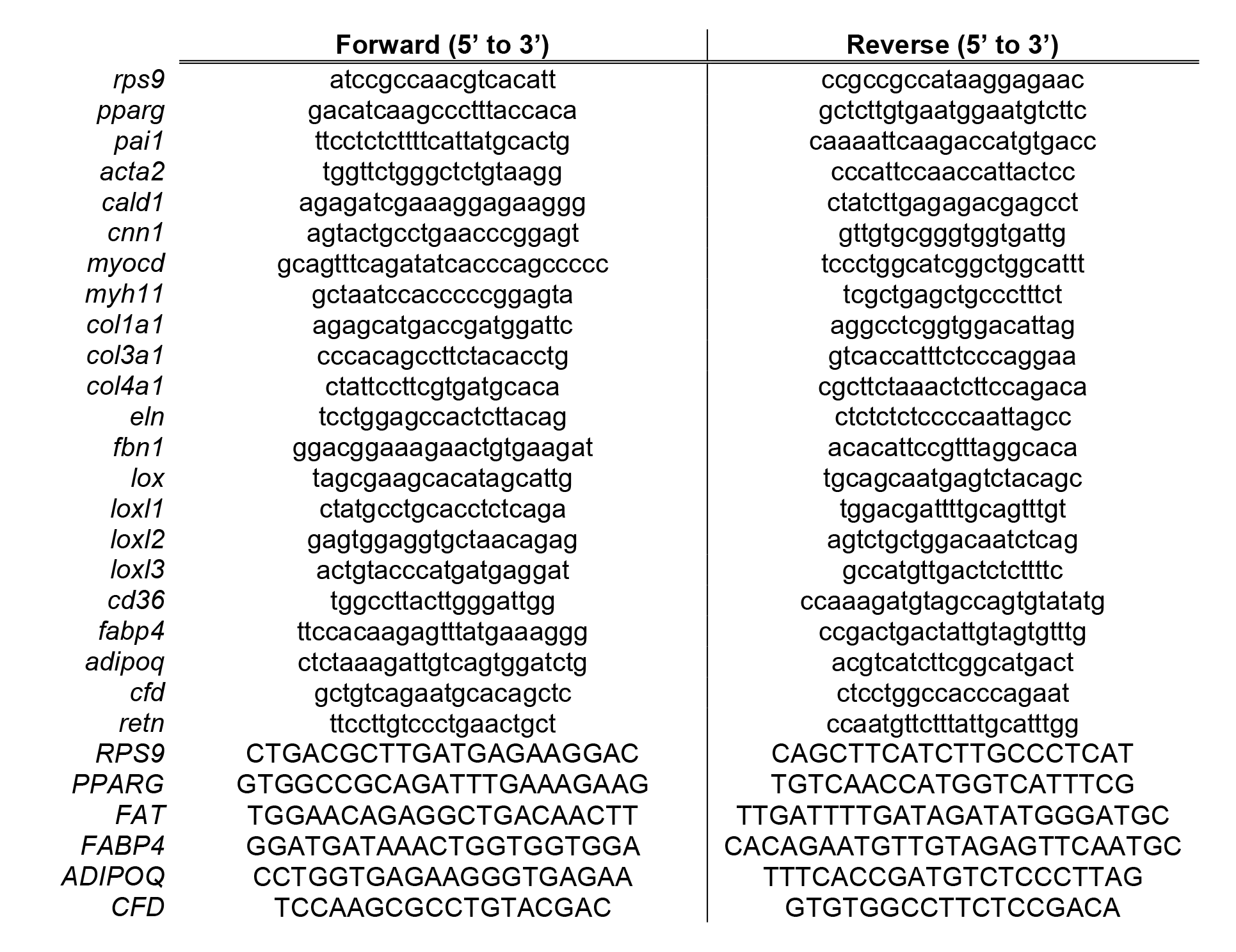

Supplement: S1 Table — Mouse primers in lowercase. Human primers in uppercase. (TIF) [file pone.0139756.s001.tif]

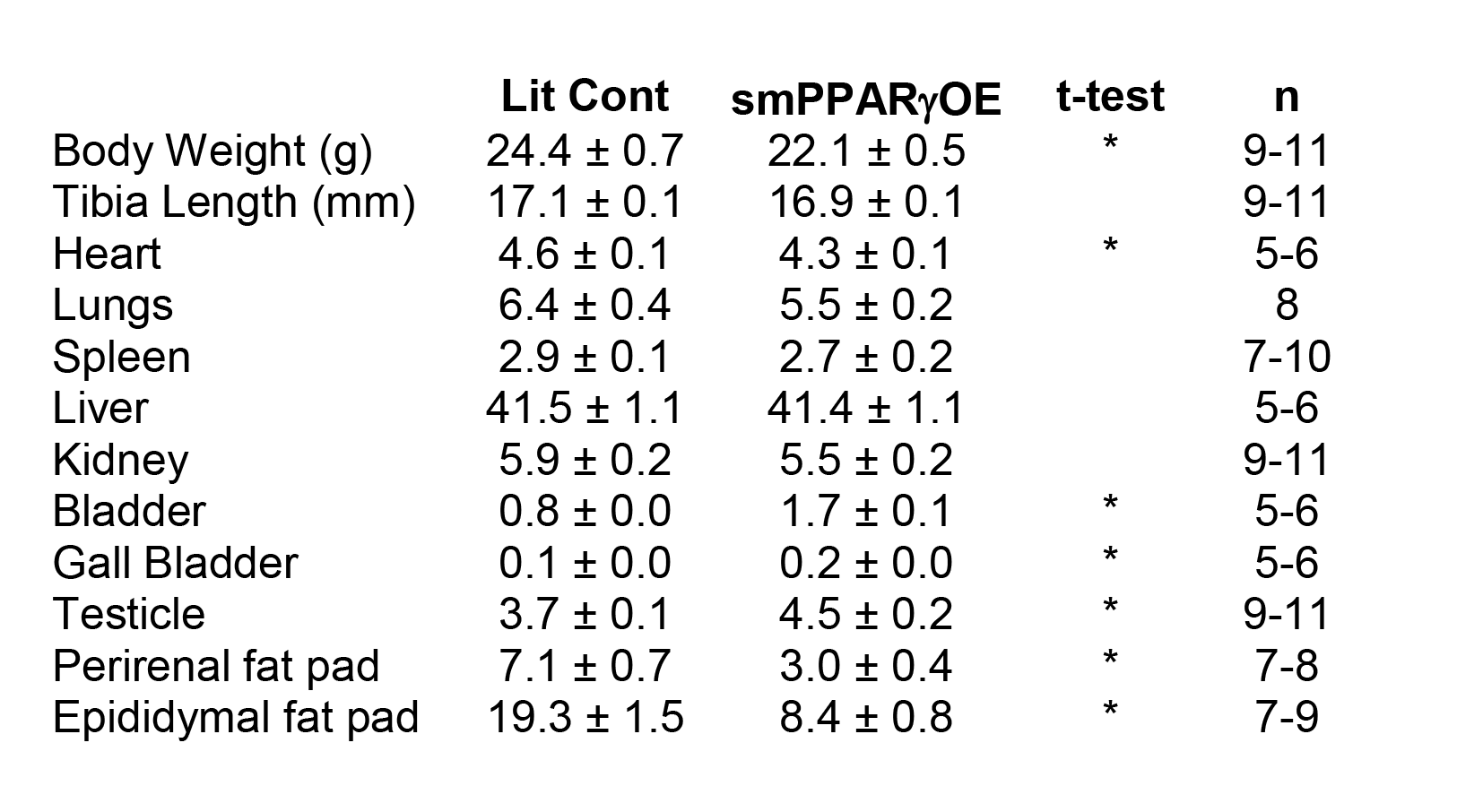

Supplement: S2 Table — Selected organs and tissues were harvested from 11 week-old mice (5 weeks after tamoxifen injection) and weighed or measured. Organ and fat pad measurements are presented as organ weight (mg) relative to total body weight (g) ± SEM. Kidney and testicle data are presented as pair average (mg) / total body weight (g). *p<0.05 by unpaired t-test. (TIF) [file pone.0139756.s002.tif]

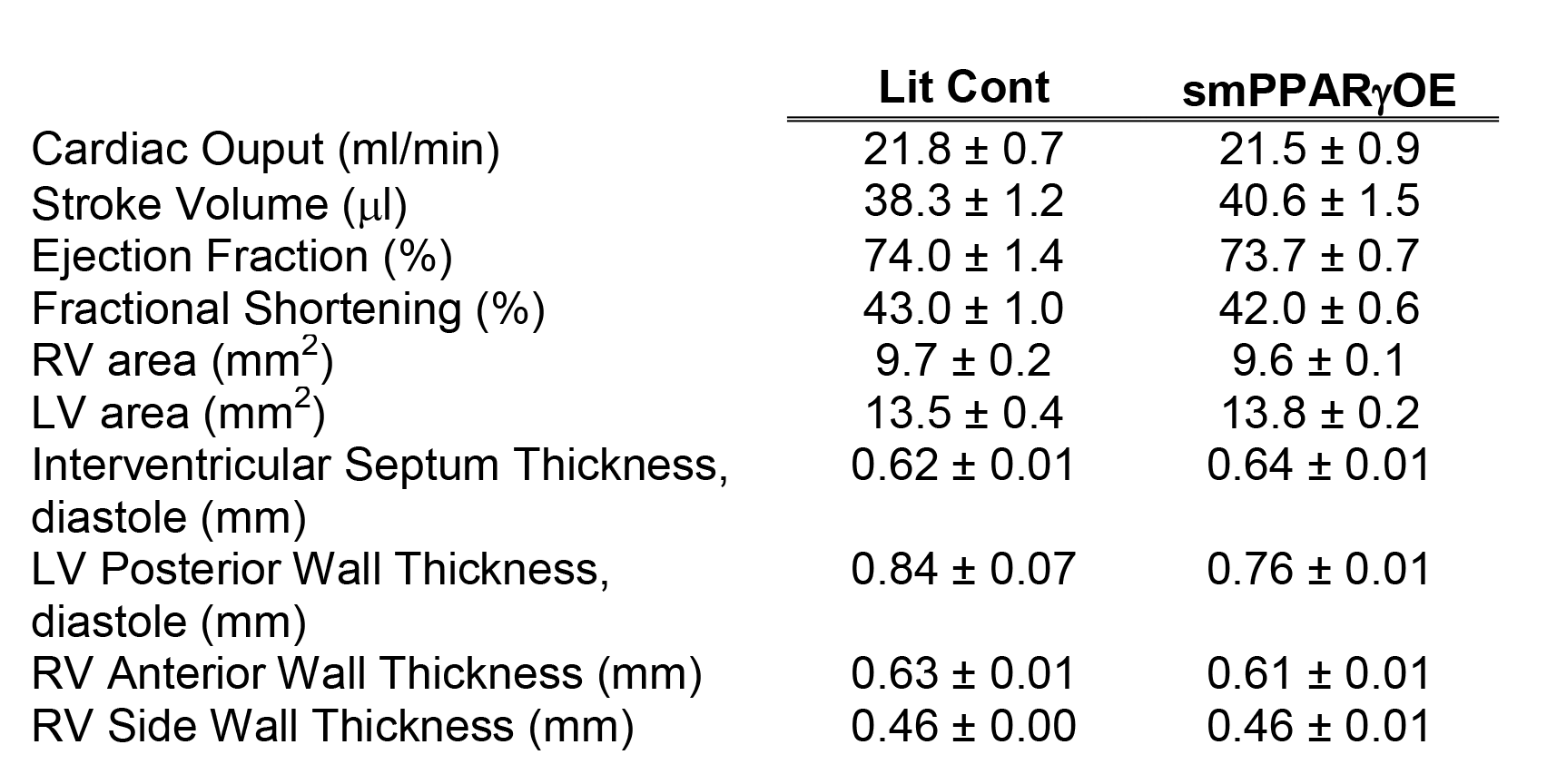

Supplement: S3 Table — Echocardiographic data from littermate control (Lit Cont) and smPPARγOE mice at 4 weeks post-tamoxifen. Values represent mean ± SEM from 7 animals. Unpaired t-tests revealed no significant differences between Lit Cont and smPPARγOE mice. (TIF) [file pone.0139756.s003.tif]

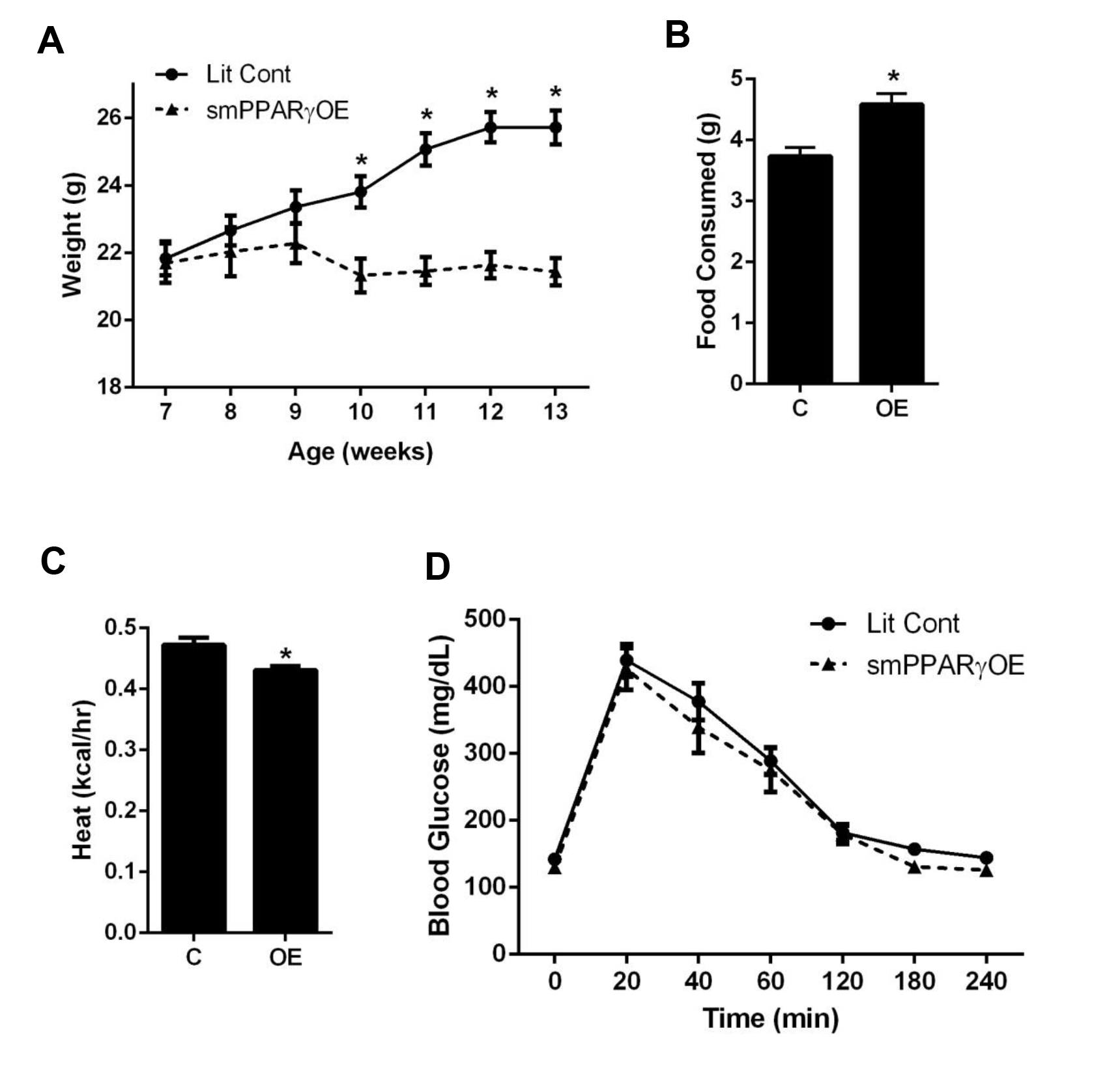

Supplement: S1 Fig — 6 week old littermate control (Lit Cont or C) and smPPARγOE (OE) mice were injected with tamoxifen (50 mg/kg/day for 5 days). Body weights of 13 mice were tracked over time (A). Data points represent mean ± SEM body mass in grams. *p<0.05 using 2-way ANOVA with repeated measures and Sidak’s post-test. Mice (14 weeks after tamoxifen-induced recombination) were housed singly, and food consumption was observed over 24 hours (B). Each bar represents the mean ± SEM food consumed in grams from 5–6 animals. *p<0.05 using unpaired t-test. Mice were then placed in an Oxymax Lab Animal Monitoring System to determine the volume of O2 consumed and CO2 produced. Volumes were measured for 60 seconds and sampled every 30 minutes for 24 hours. Energy expenditure (heat) was calculated as CV*VO2, where CV = 3.815+[1.232*(VCO2/VO2)]. The data over 24 hours were averaged and graphed in (C). Each bar represents the group mean ± SEM heat (kcal/hr) from ten mice. *p<0.05 using unpaired t-test. In (D), intraperitoneal glucose injections were administered to Lit Cont and smPPARγOE mice four week post-tamoxifen, and serum glucose values were determined with an Accu-check Aviva glucose meter at intervals for 4 hours. Data points represent the mean ± SEM blood glucose in mg/dL from 9 mice. (TIF) [file pone.0139756.s004.tif]

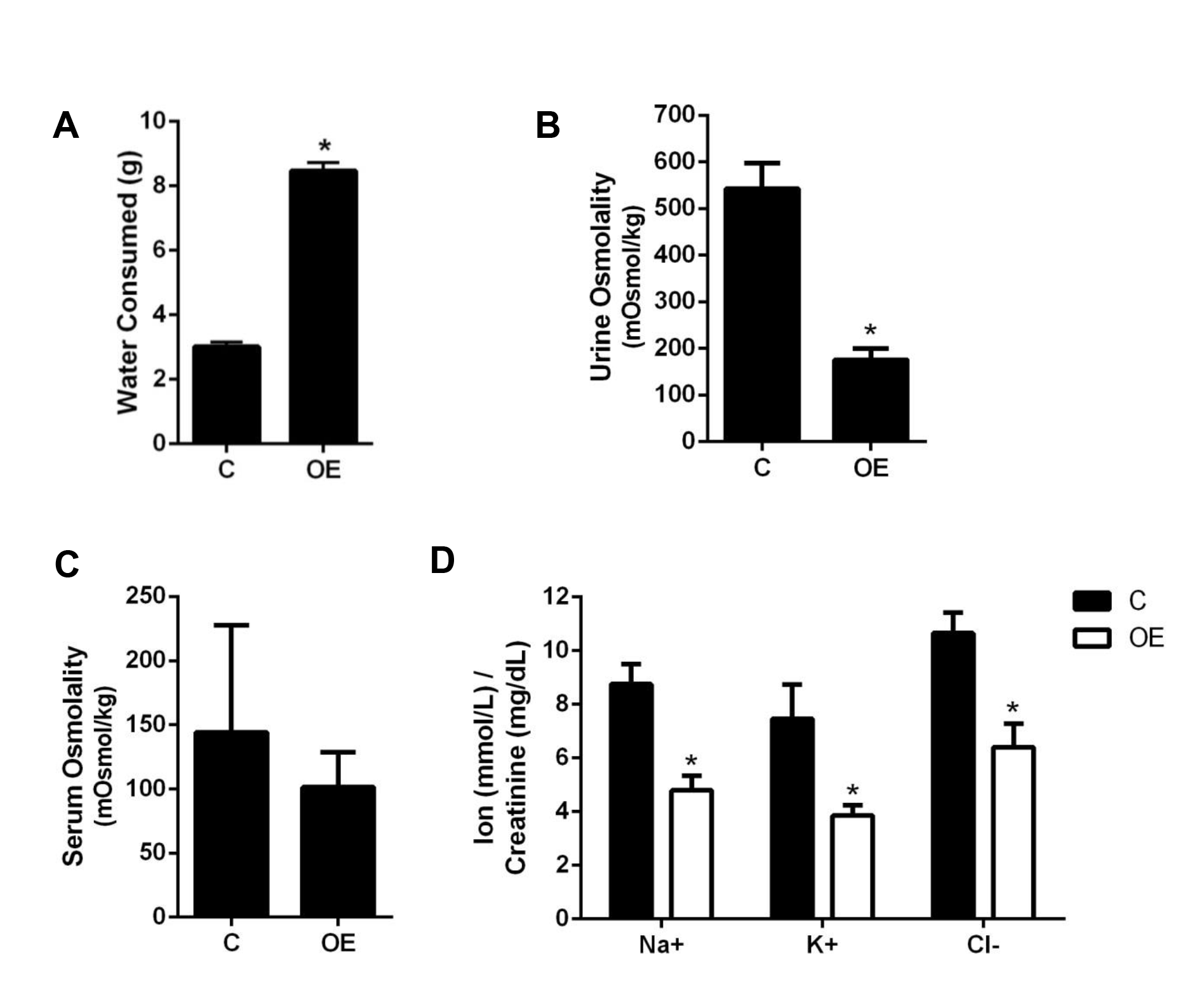

Supplement: S2 Fig — In (A), 14-weeks following tamoxifen-induced recombination, littermate control (C) and smPPARγOE (OE) mice were housed singly and water consumption was measured for 24 hours. Each bar represents the mean ± SEM water consumed (grams) per mouse from 5–6 mice. After sacrifice, urine and blood samples were collected simultaneously. Urine (B) and serum (C) osmolality were measured using an osmometer in mice following ad lib water intake. Bars represent the mean ± SEM osmolality (mOsmol/kg) from 3–5 animals. In (D), urine Na+, K+, and Cl- levels were measured and expressed relative to urine creatinine levels. n = 3–4. For all, *p<0.05 using unpaired t-tests. (TIF) [file pone.0139756.s005.tif]

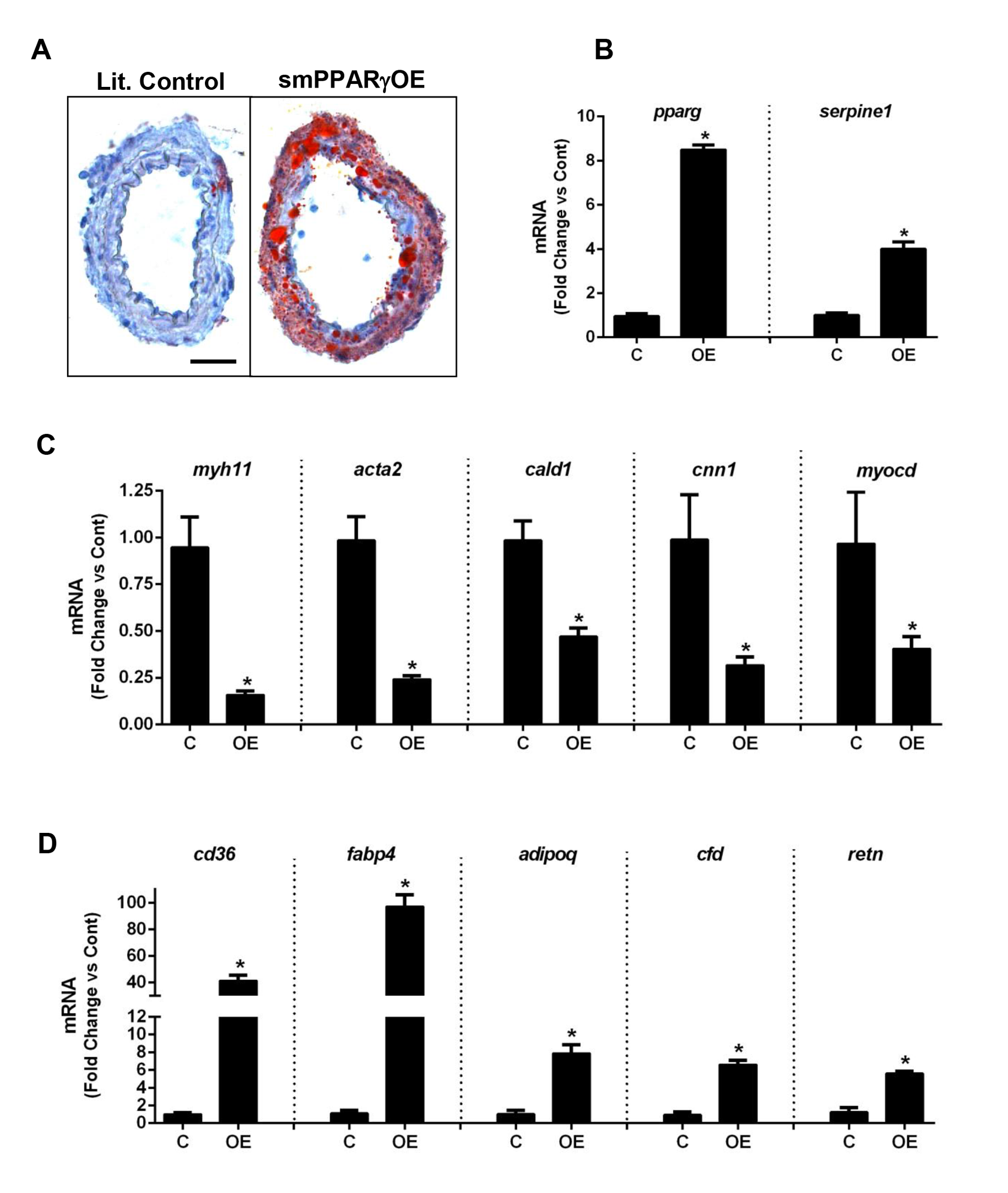

Supplement: S3 Fig — (A) Four to six weeks following tamoxifen injection, the intestinal branches of the superior mesenteric artery were isolated from littermate control and smPPARγOE mice. Vessels were cleaned of adipose tissue and frozen in OCT blocks. Cross sections were stained with an oil red O kit. Sections were examined at 20x with light microscopy. Red = lipid. Scale bar = 35 µm. Separately, mesenteric arteries were collected, pooled (two per sample), and RNA isolated. qRT-PCR was performed. pparg and downstream target gene serpine1 are shown in (B). Contractile proteins are displayed in (C) and adipocyte-related markers are displayed in (D). Each bar represents the mean ± SEM copies of mRNA normalized to rps9 in the same sample and expressed as fold change vs C. n = 4. *p<0.05 by unpaired t-tests. (TIF) [file pone.0139756.s006.tif]

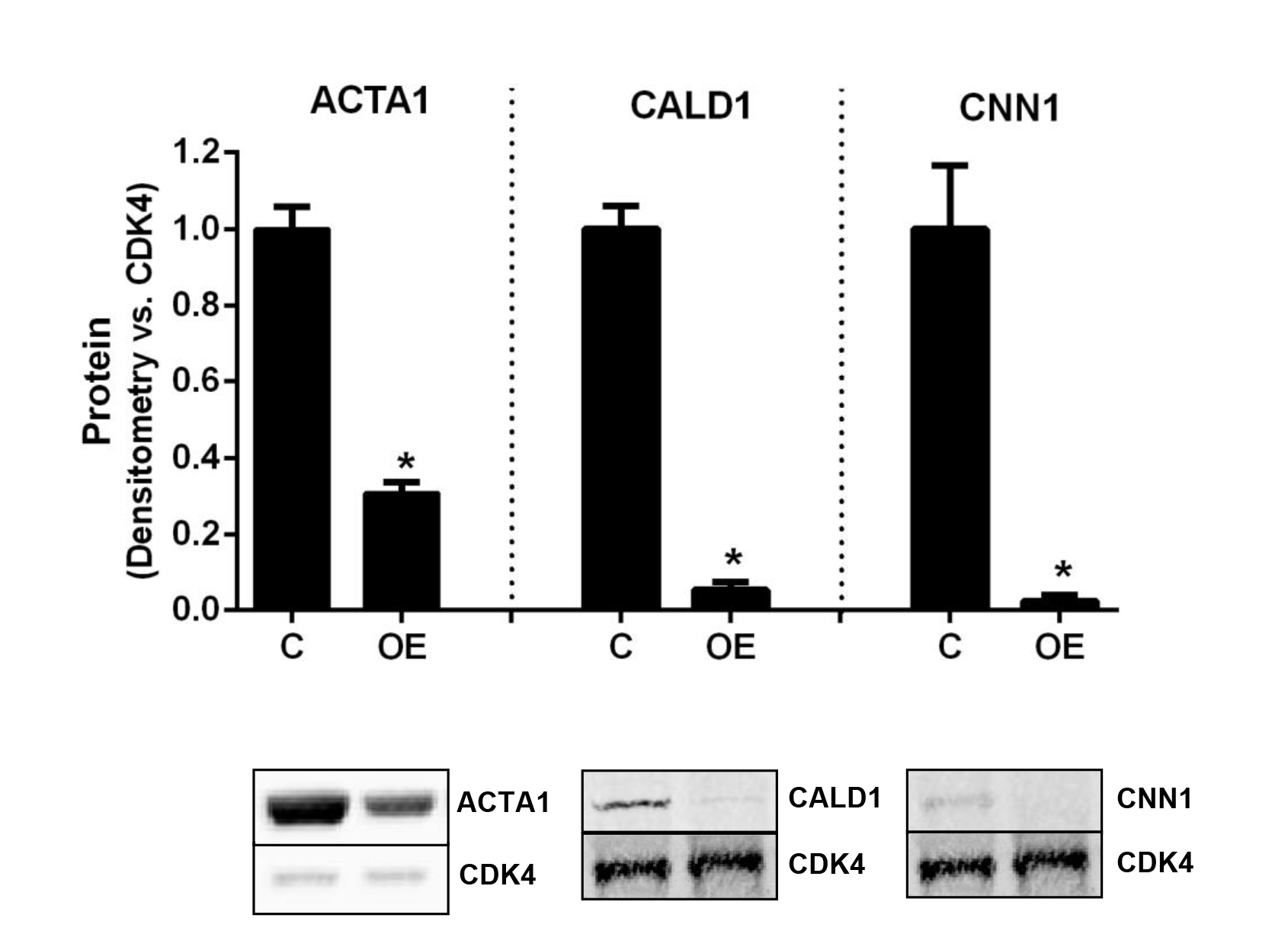

Supplement: S4 Fig — Alpha smooth muscle actin (ACTA1), caldesmon (CALD1), and calponin (CNN1) protein levels were determined by western blot and densitometry in whole aortic lysates. Each bar represents the mean ± SEM protein densitometric intensity relative to CDK4 and expressed as fold change vs control for 3–5 animals at 3–11 weeks post-tamoxifen. Representative blots are presented below the graphs. *p<0.05 using unpaired t-test. (TIF) [file pone.0139756.s007.tif]

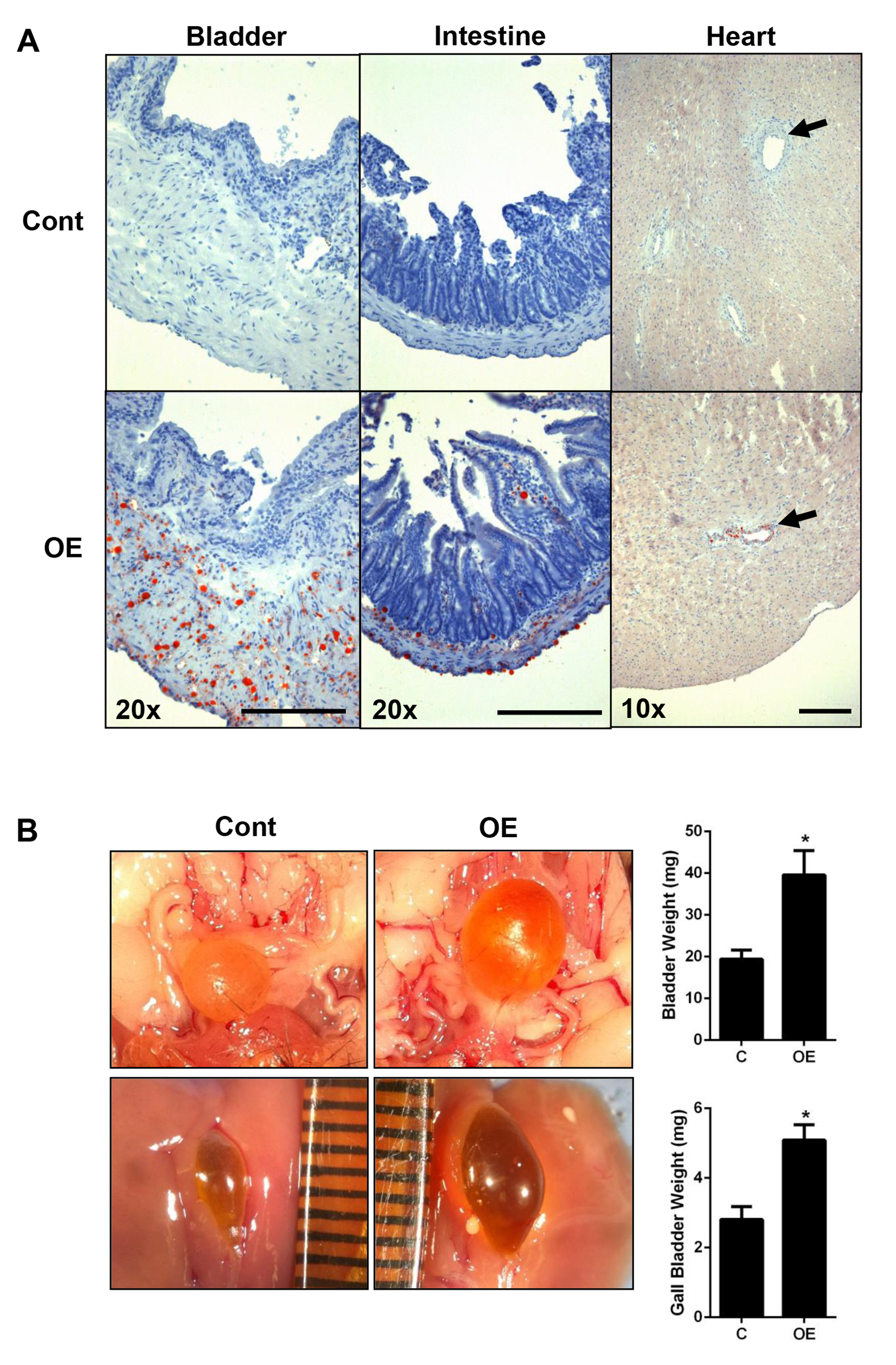

Supplement: S5 Fig — In (A), frozen sections from the bladder, intestine, and heart of littermate control (C or Cont) and smPPARγOE (OE) mice were stained with oil red O. Representative images are shown. Arrow = large artery. Scale bars = 100 μm. In (B), representative images of the urinary bladder (top) and gall bladder (bottom) from LC and OE mice are presented. The bladders were then removed, drained, and weighed. Each bar represents the mean ± SEM urinary or gall bladder weight from 5–6 mice, four weeks post-tamoxifen. *p<0.05 using unpaired t-test. (TIF) [file pone.0139756.s008.tif]

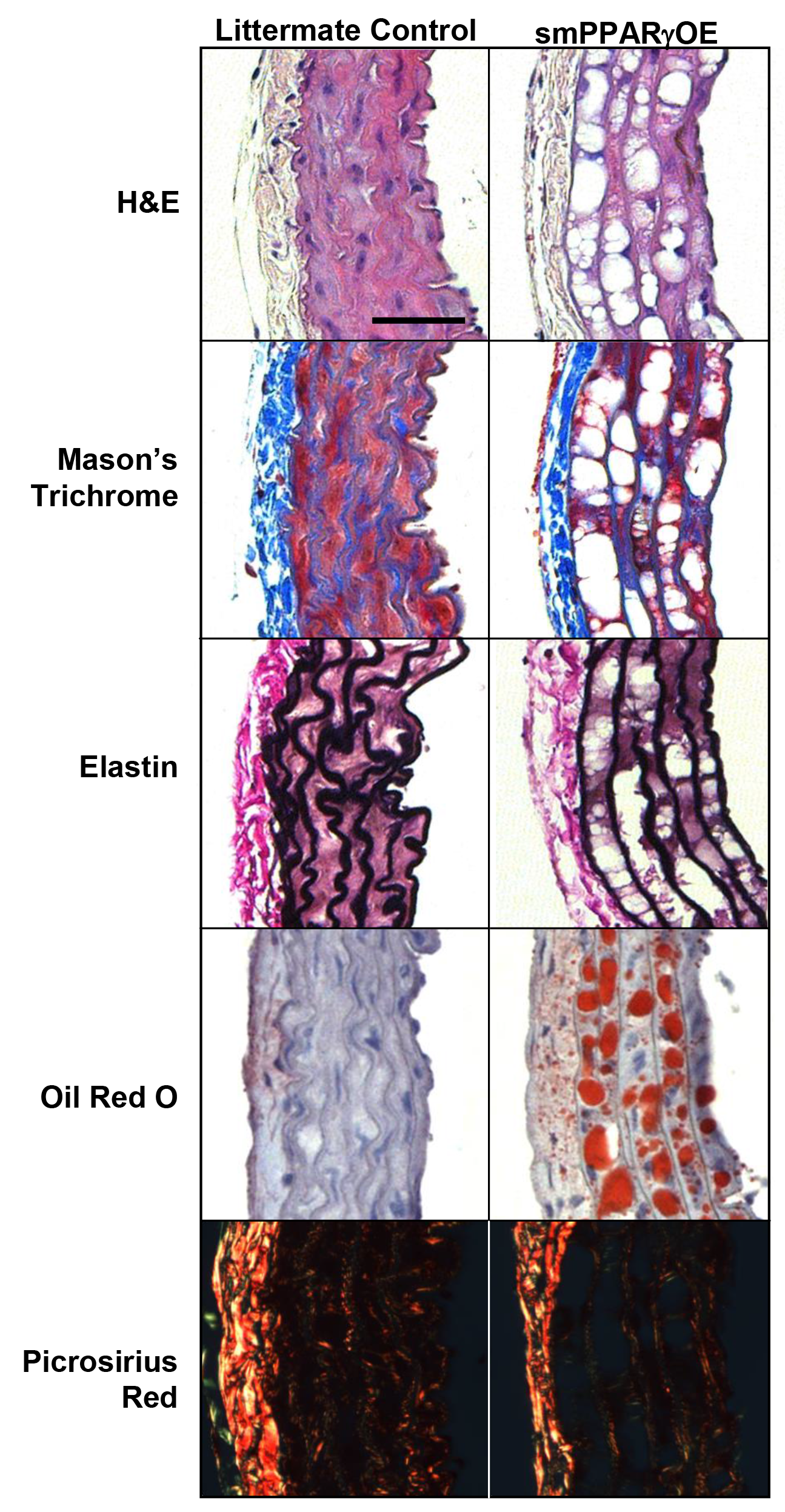

Supplement: S6 Fig — 28-weeks following tamoxifen-induced recombination, the descending aortas from littermate control and smPPARγOE mice were isolated, fixed, and embedded in paraffin (except oil red O stain which utilized frozen tissue blocks). Cross sections were stained with H&E, Mason’s trichrome, elastin, oil red O, or picrosirius red stains. The resulting sections were examined at 20x using light microscopy or polarized light microscopy (picrosirius red). Representative images are presented. Scale bar = 50 μm. (TIF) [file pone.0139756.s009.tif]

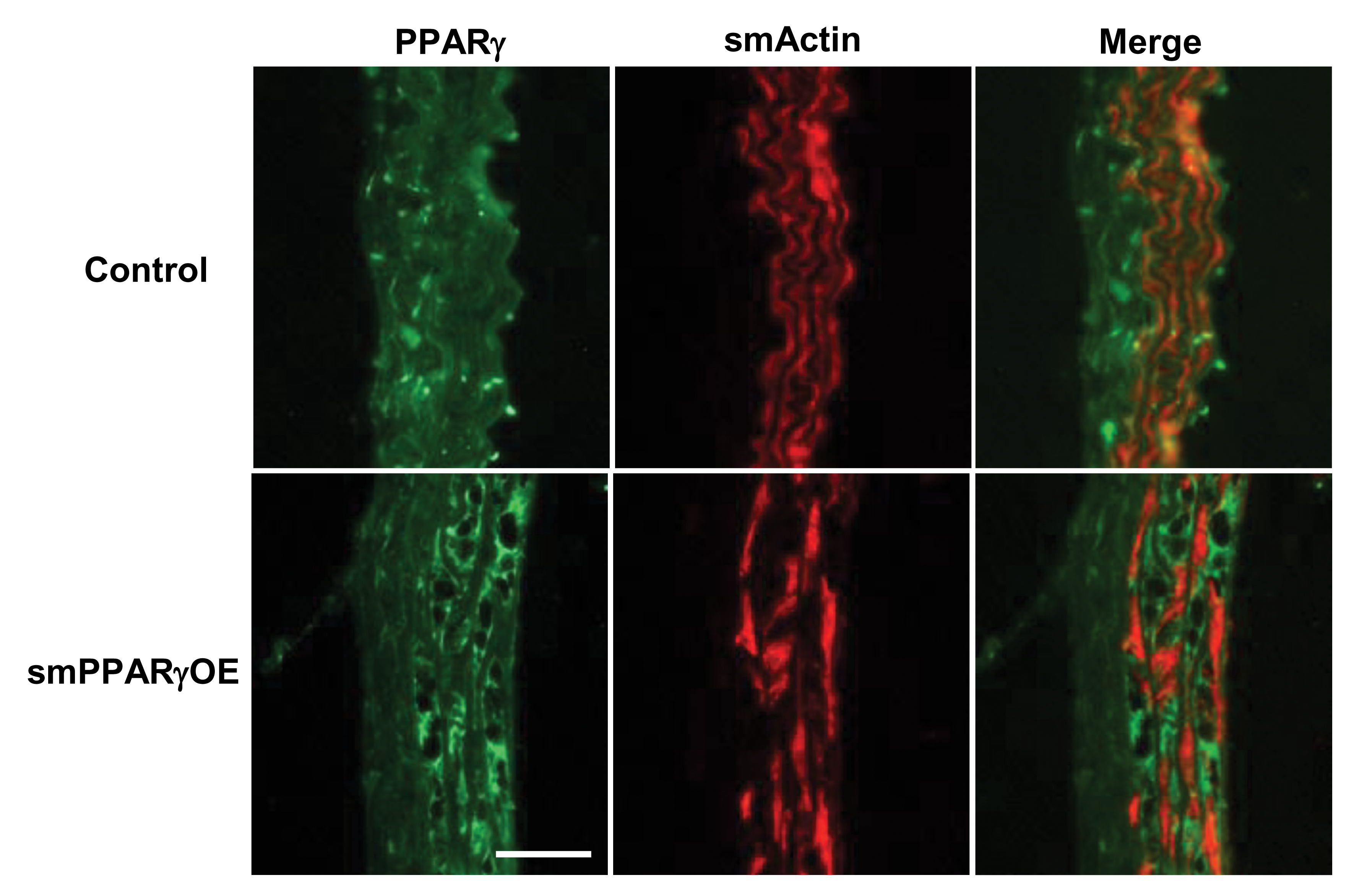

Supplement: S7 Fig — 8-weeks following tamoxifen-induced recombination, the descending aortas from littermate control and smPPARγOE mice were embedded in Optimal Cutting Temperature medium and frozen. 5μm cross sections were blocked and incubated with PPARγ (Santa Cruz sc-7273, mouse monoclonal, 1:50) and smooth muscle actin (Thermo Fisher RB-9010-P, rabbit polyclonal, 1:50) antibodies. The PPARγ signal was fluorescently labeled using a mouse-on-mouse fluorescein kit (Vector Laboratories FMK-2201). The smooth muscle actin was visualized with a rhodamine red-labeled anti-rabbit antibody (1:100). The resulting slides were examined under fluorescence at 20x. Representative images are presented from an n = 4. Scale bar = 50 μm. (TIF) [file pone.0139756.s010.tif]

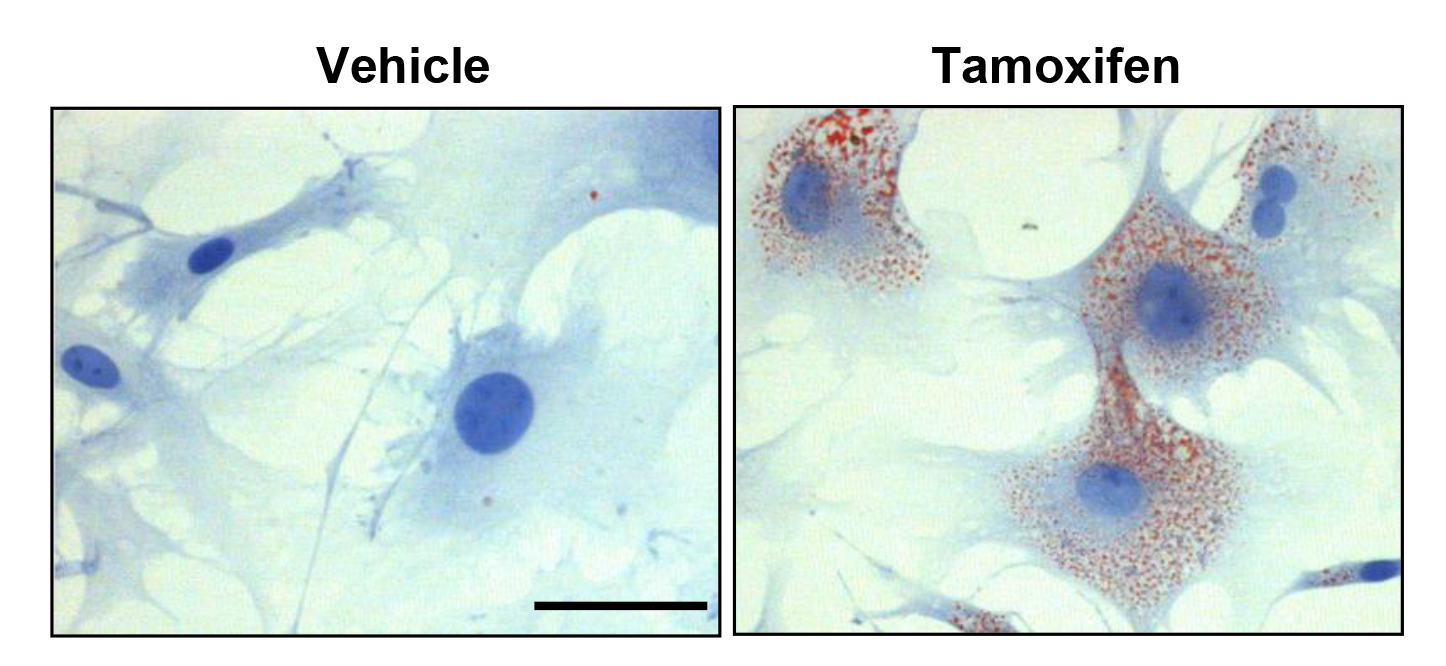

Supplement: S8 Fig — Aortas from non-induced smPPARγOE mice were digested with collagenase, and smooth muscle cells were cultured onto chamber slides. Cells were treated with vehicle (0.01% ethanol) or tamoxifen (1 μg/ml) daily for 5 days. On day 14, cells were fixed with 10% formaldehyde and stained with oil red O. Representative images are shown at 40x. Scale bar = 50 μm. (TIF) [file pone.0139756.s011.tif]

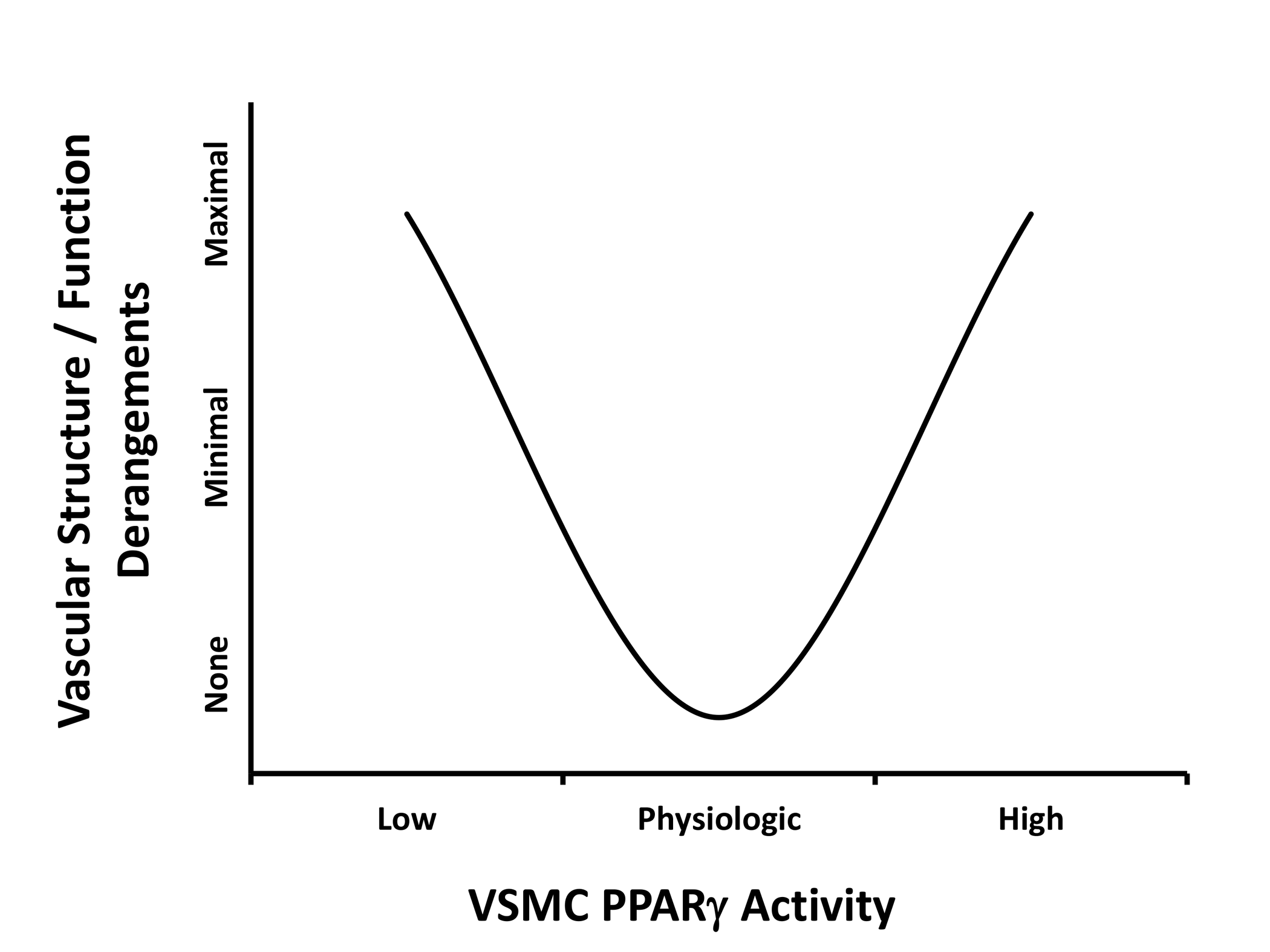

Supplement: S9 Fig — Current evidence indicates that both low and high levels of PPARγ activity in VSMC perturb vascular homeostasis suggesting that intermediate levels are required for normal vascular function and that therapeutic targeting of vascular PPARγ activity might enable reductions in selected pathophysiological derangements in vascular function caused by altered PPARγ activity. (TIF) [file pone.0139756.s012.tif]

## Slide 1
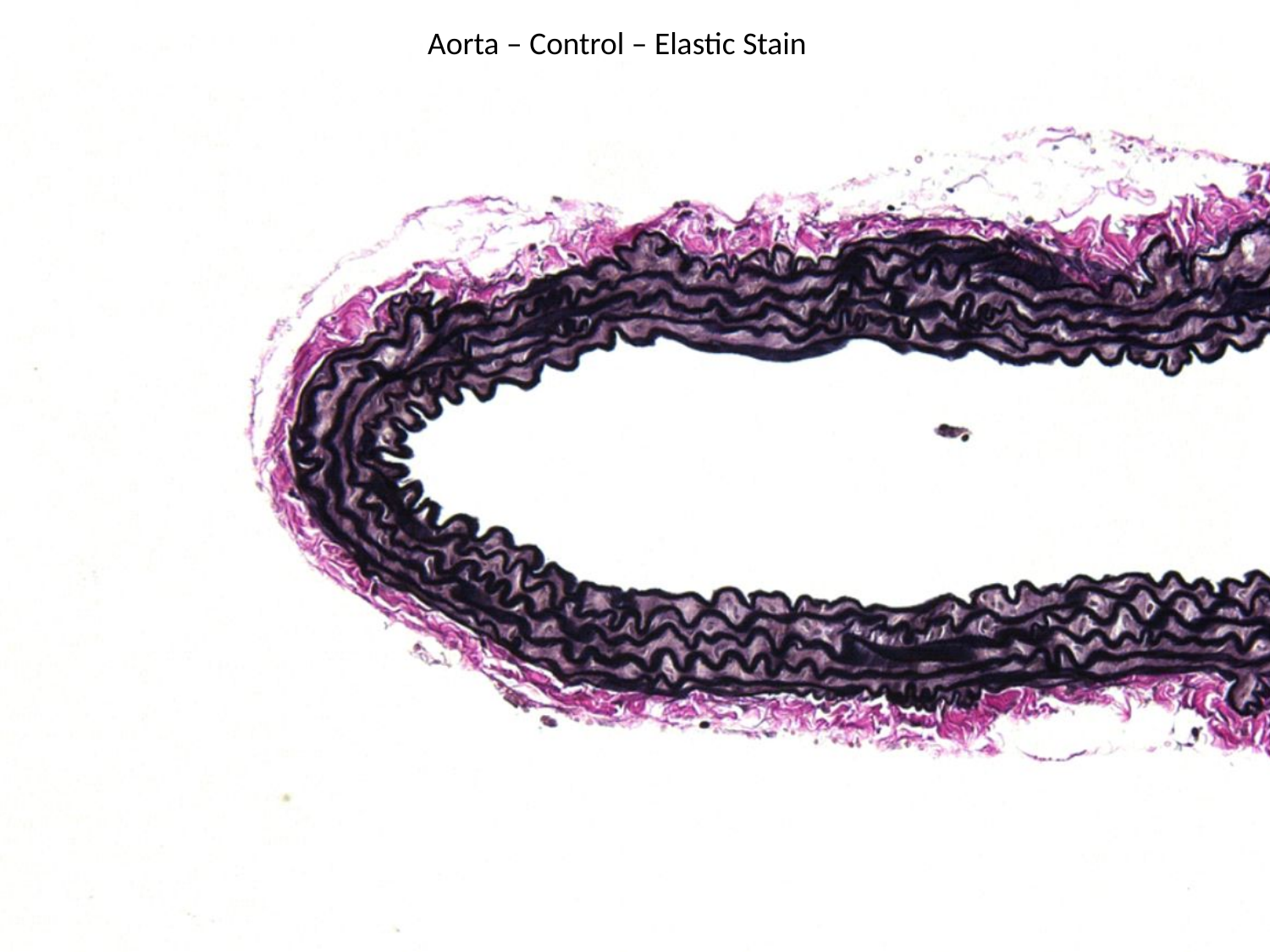

Aorta – Control – Elastic Stain

## Slide 2
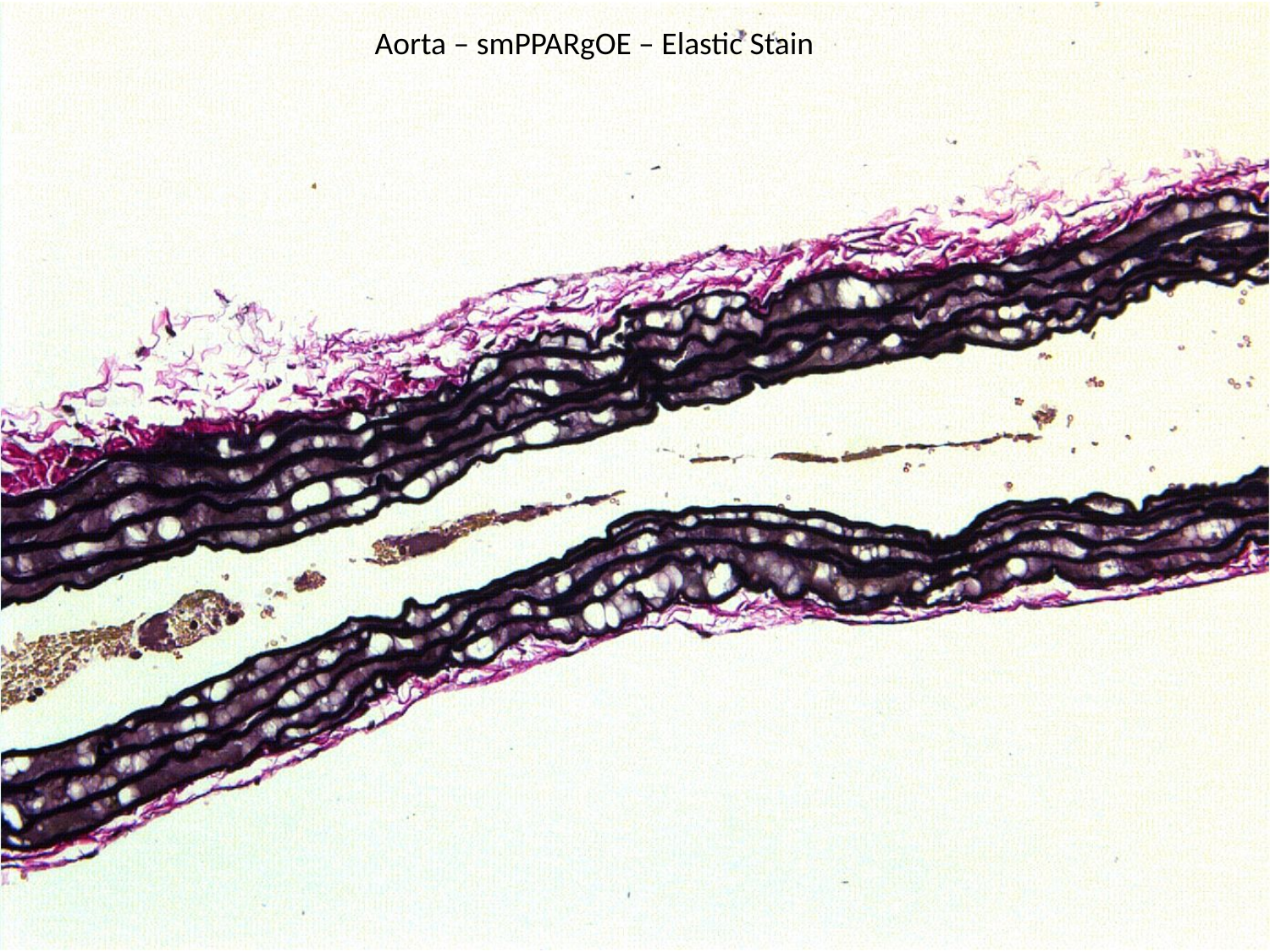

Aorta – smPPARgOE – Elastic Stain

Supplement: S1 Raw Data — (PPTX) [file pone.0139756.s013.pptx]

## Slide 1
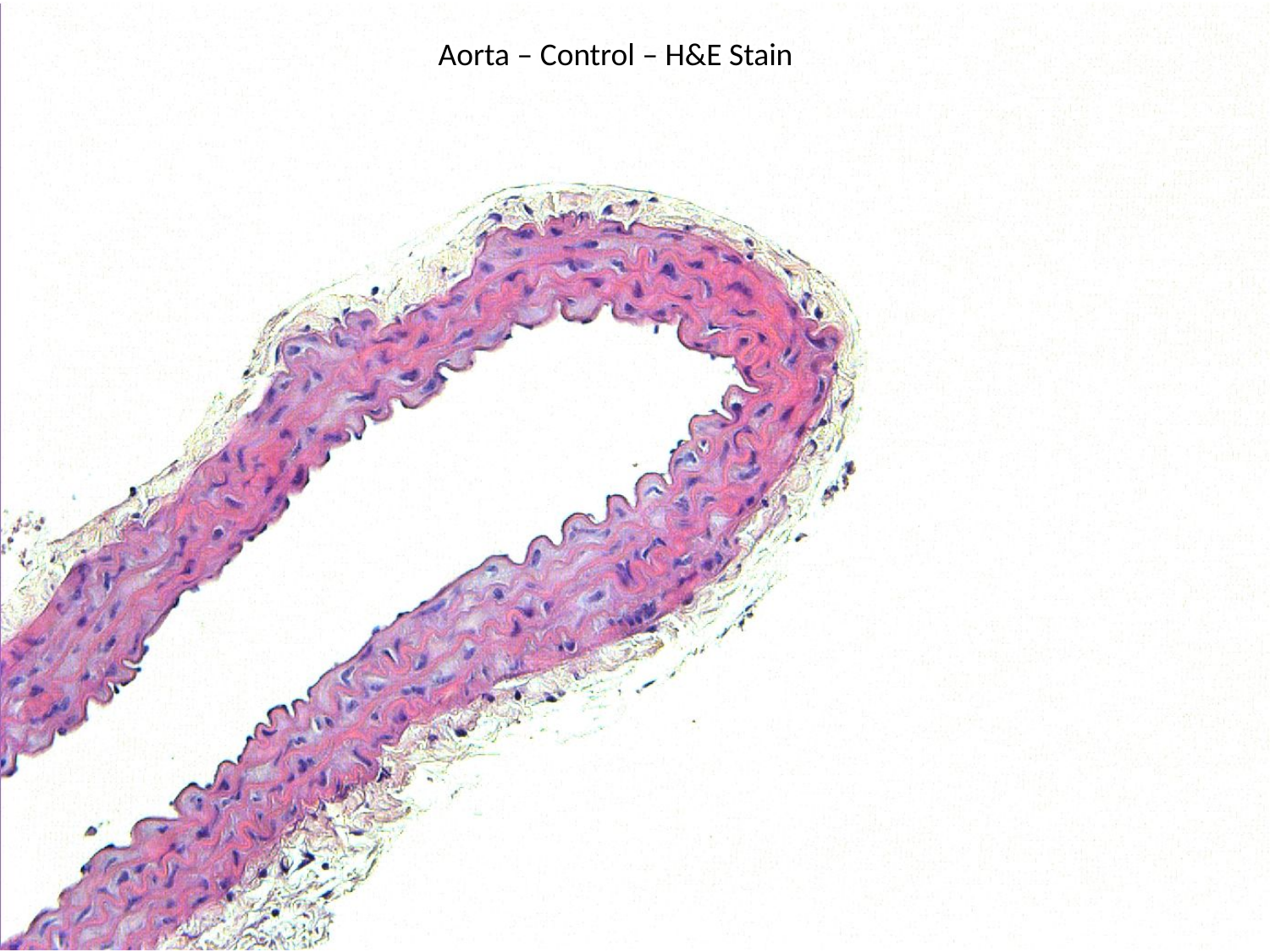

Aorta – Control – H&E Stain

## Slide 2
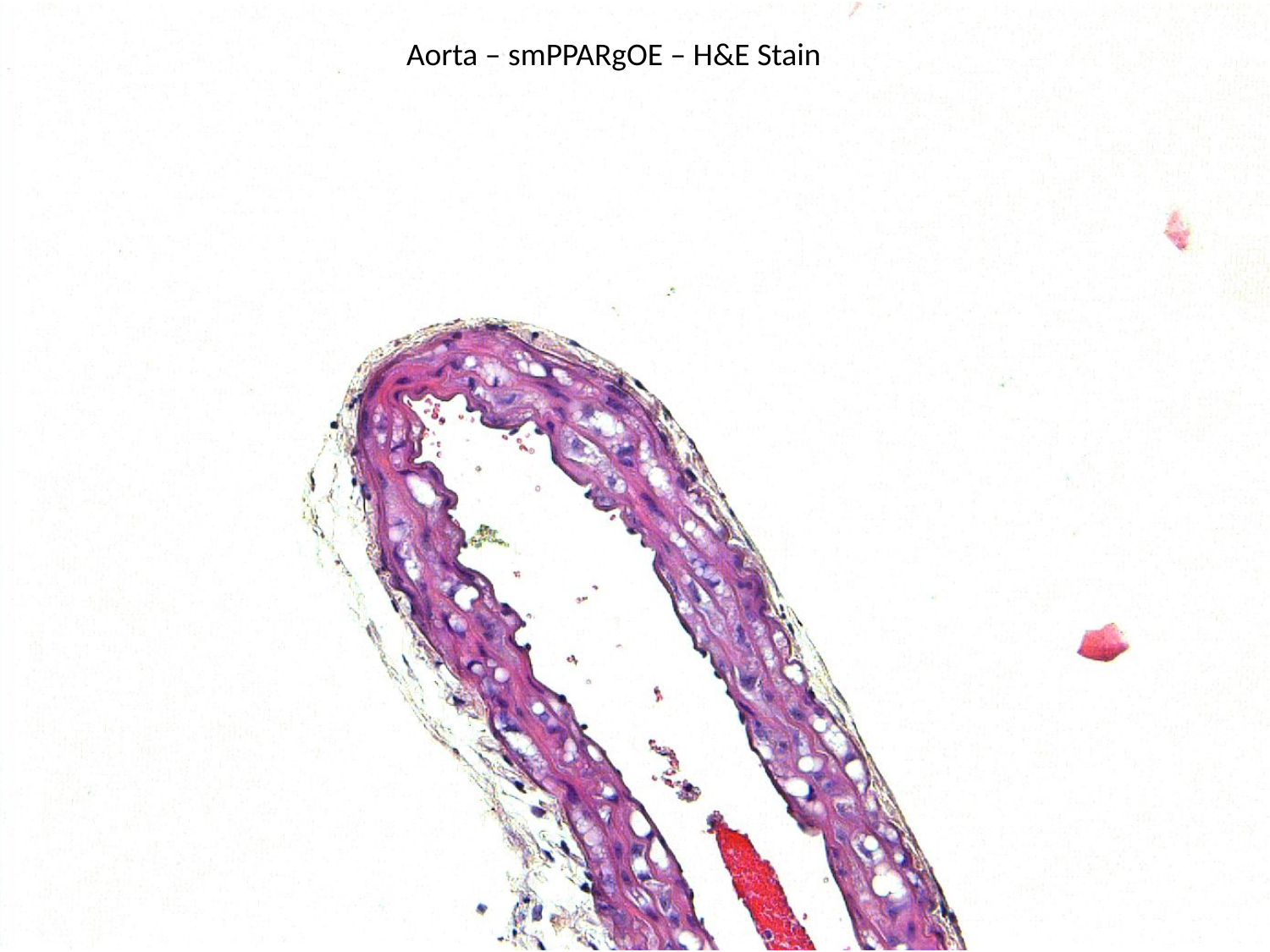

Aorta – smPPARgOE – H&E Stain

Supplement: S2 Raw Data — (PPTX) [file pone.0139756.s014.pptx]

## Slide 1
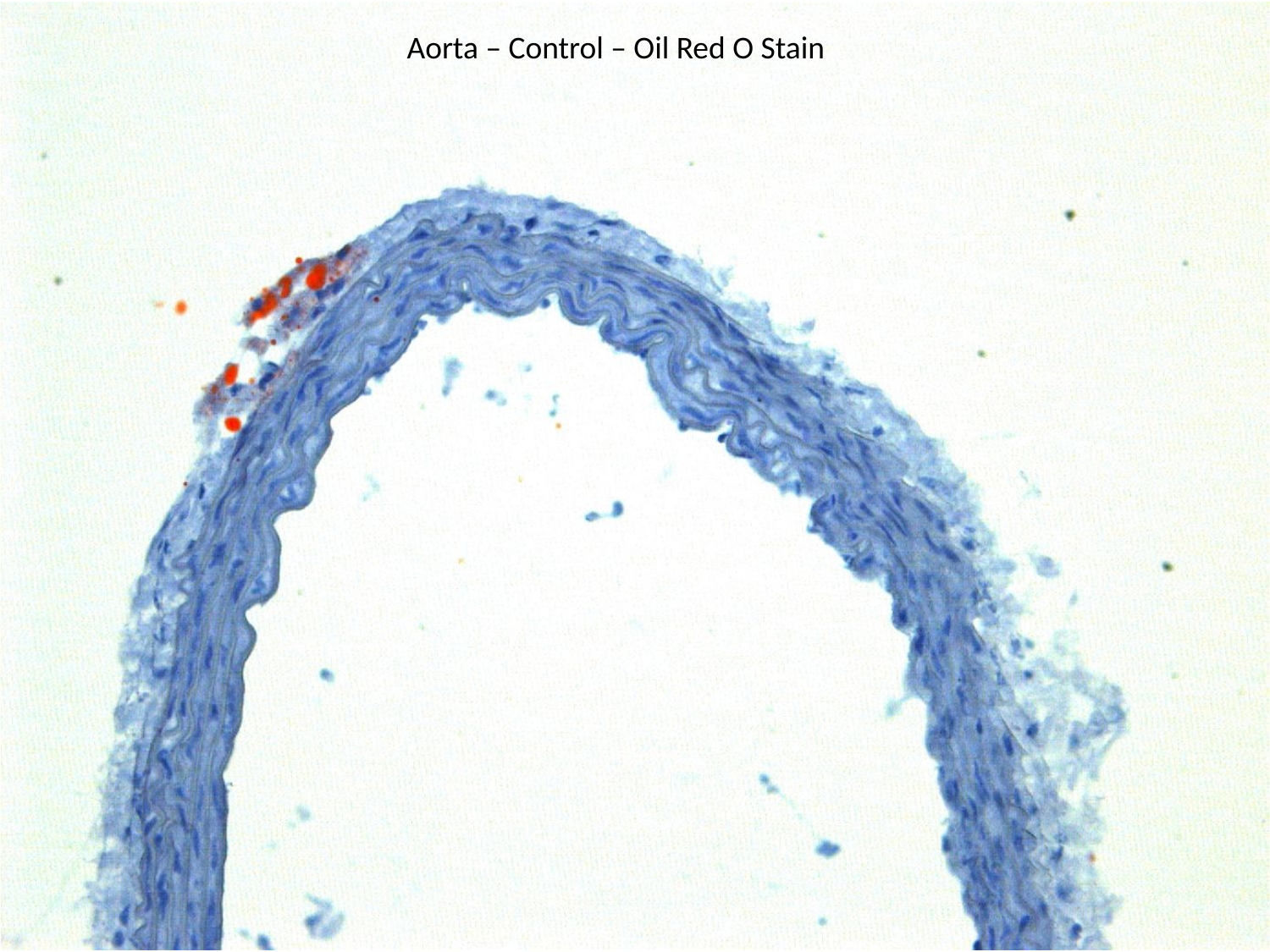

Aorta – Control – Oil Red O Stain

## Slide 2
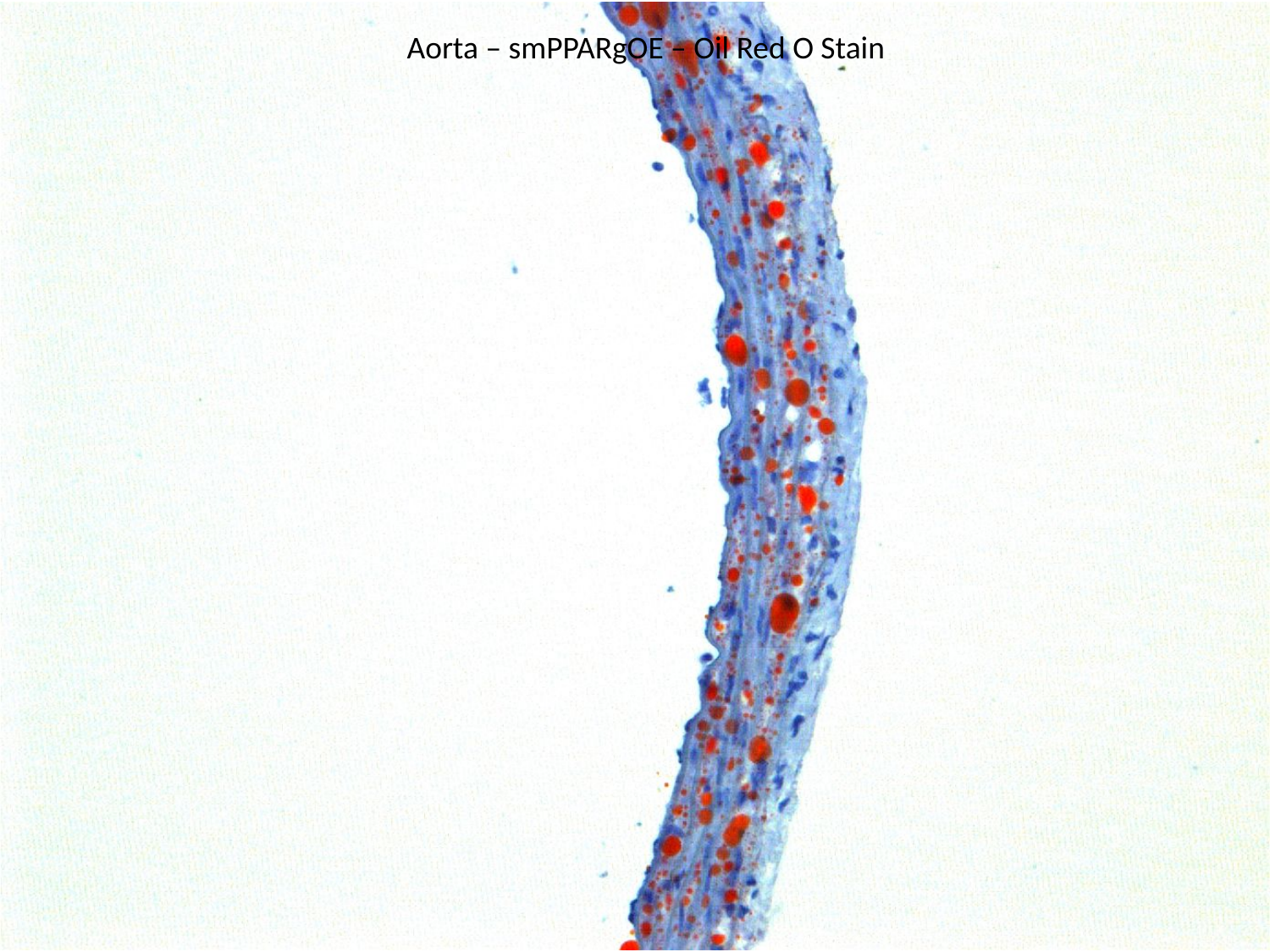

Aorta – smPPARgOE – Oil Red O Stain

Supplement: S3 Raw Data — (PPTX) [file pone.0139756.s015.pptx]

## Slide 1
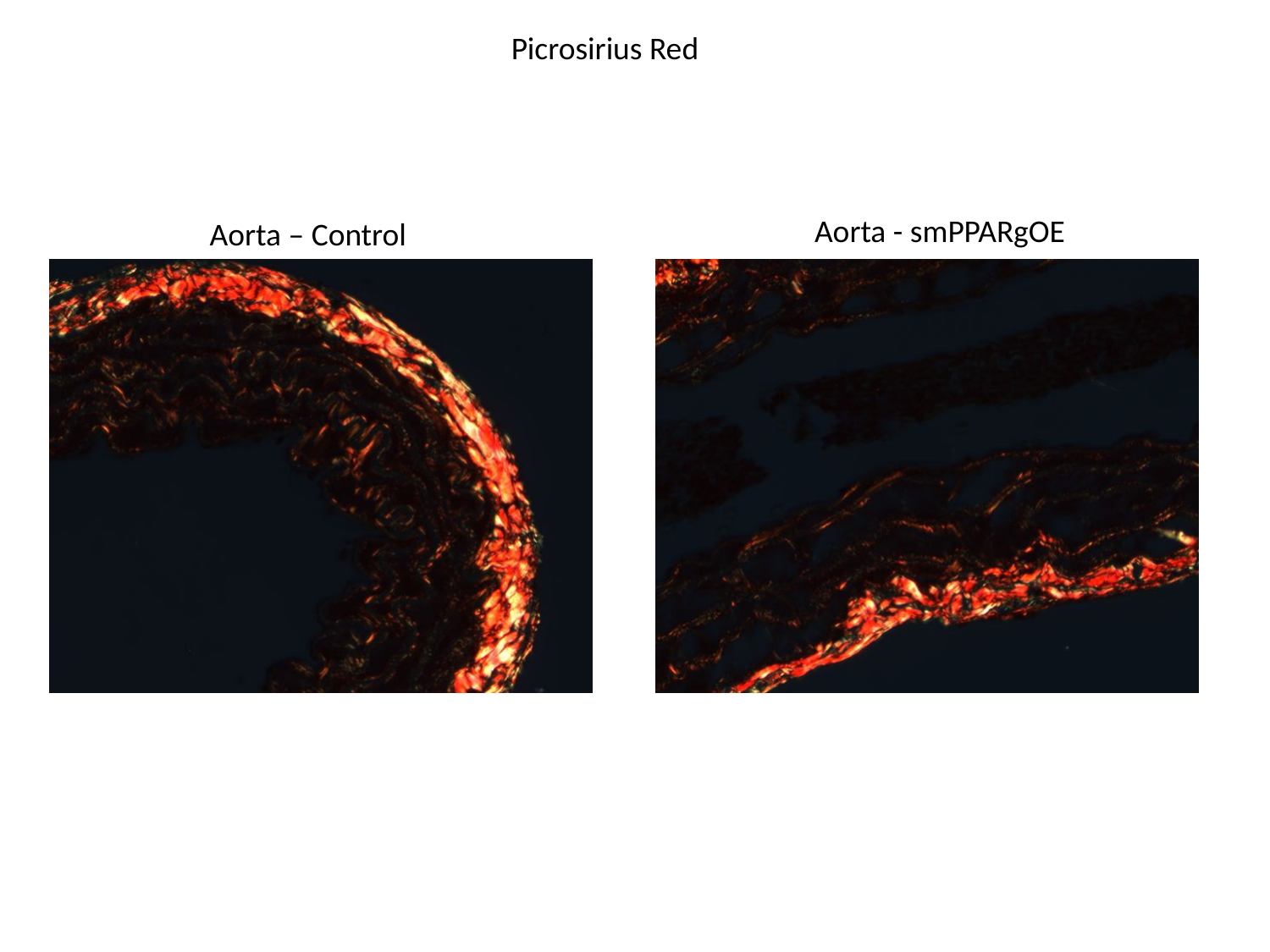

Picrosirius Red
Aorta - smPPARgOE
Aorta – Control

Supplement: S4 Raw Data — (PPTX) [file pone.0139756.s016.pptx]

## Slide 1
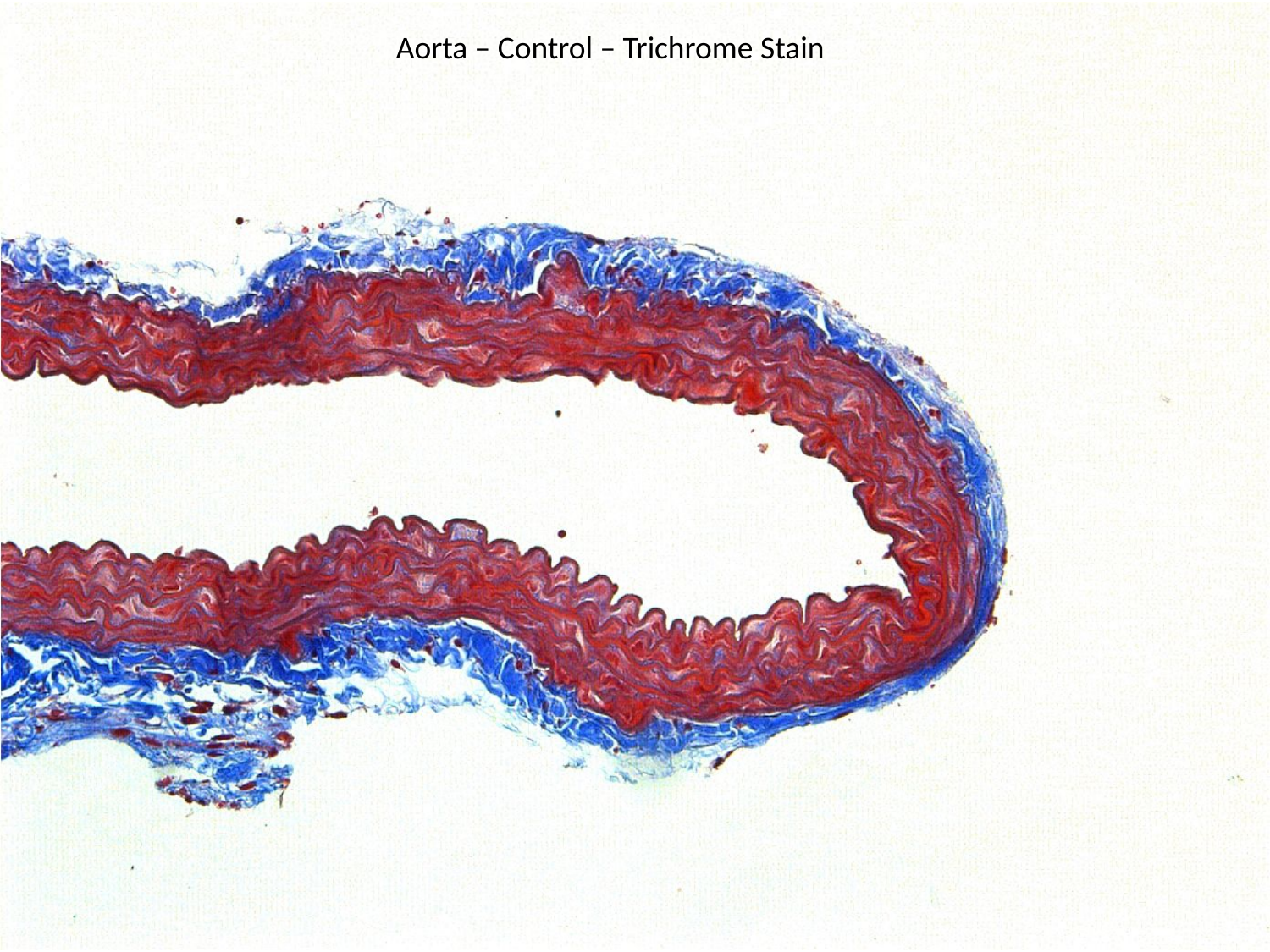

Aorta – Control – Trichrome Stain

## Slide 2
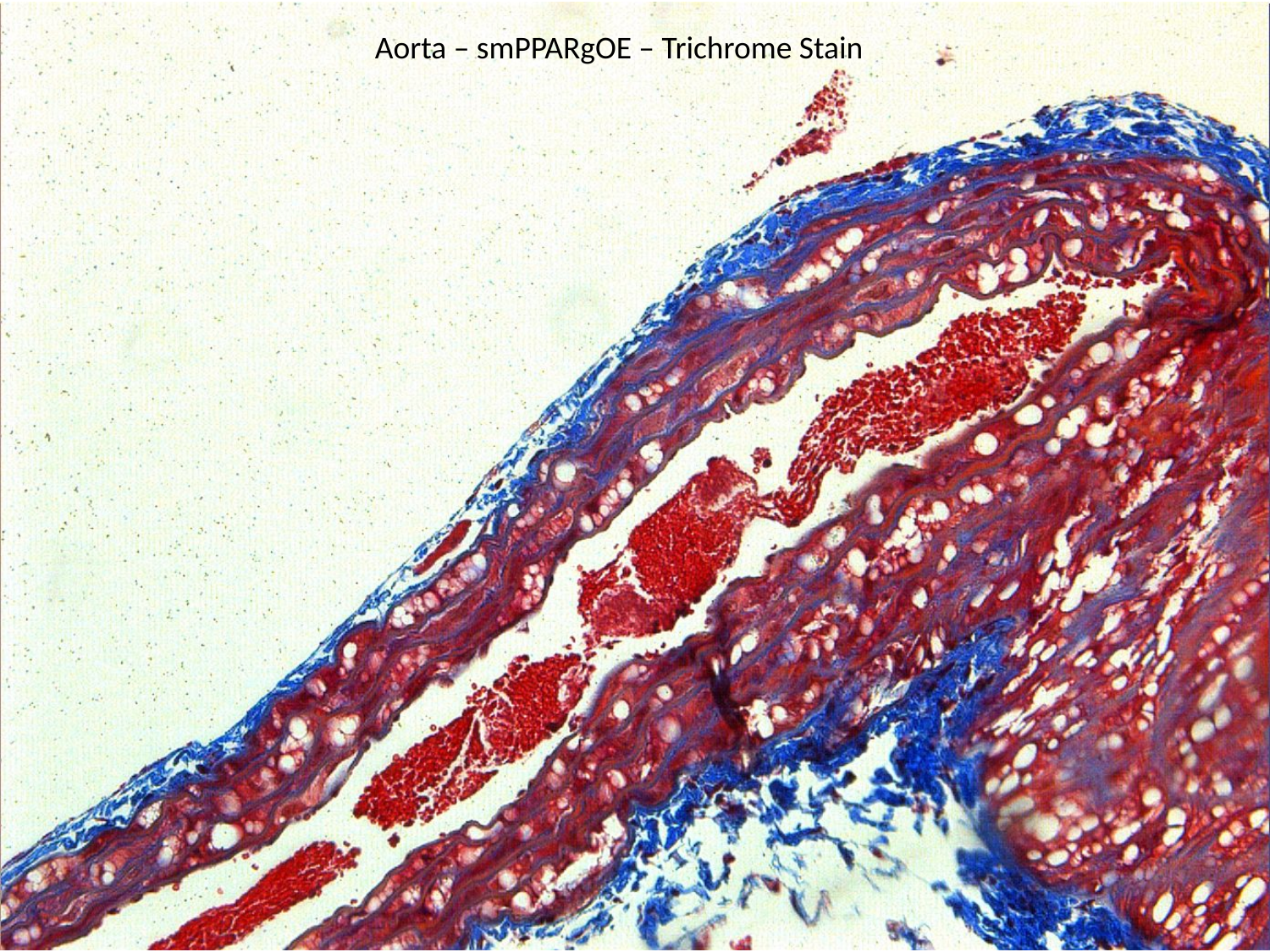

Aorta – smPPARgOE – Trichrome Stain

Supplement: S5 Raw Data — (PPTX) [file pone.0139756.s017.pptx]

## Slide 1
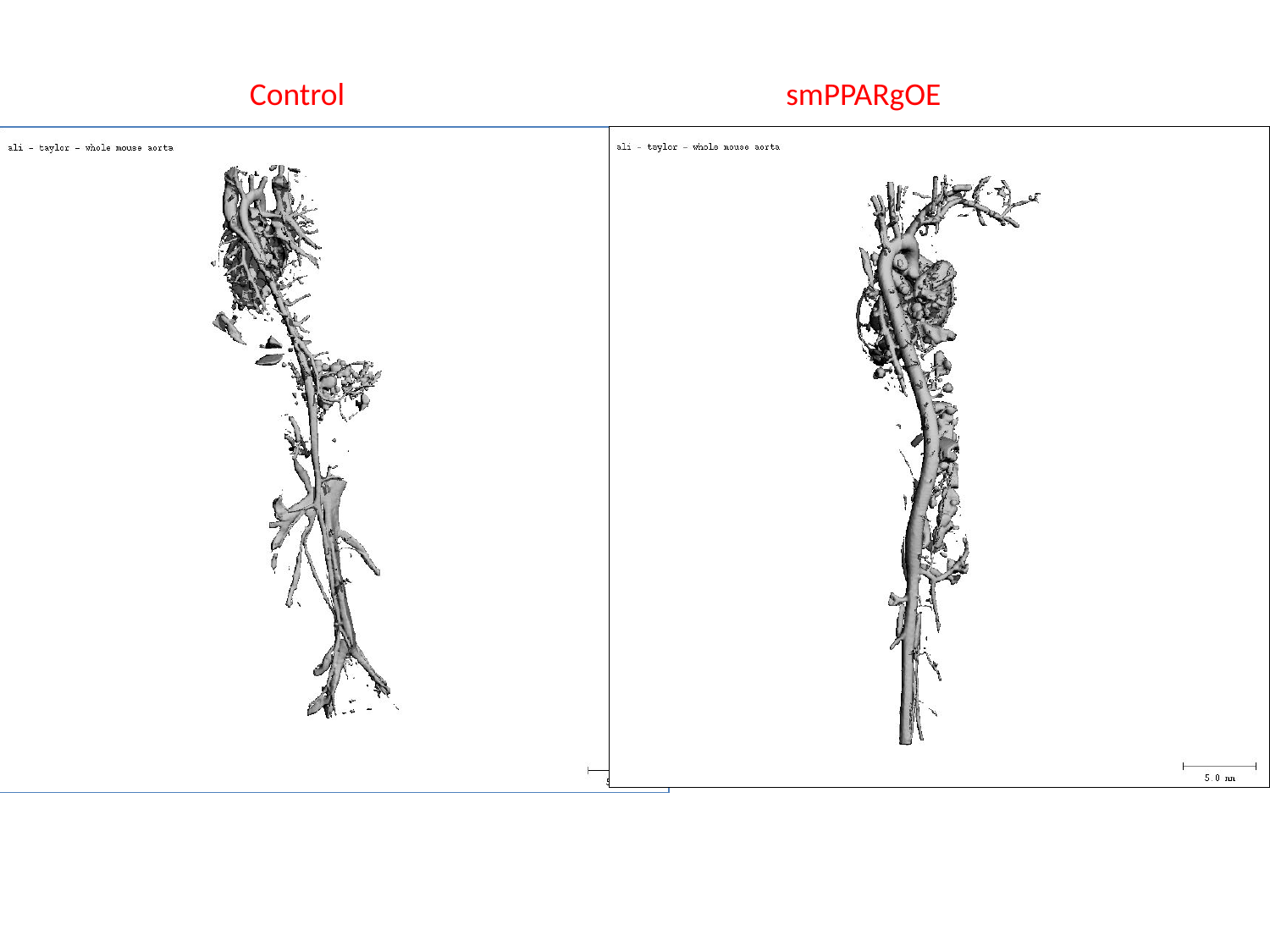

smPPARgOE
Control

Supplement: S6 Raw Data — (PPTX) [file pone.0139756.s018.pptx]

## Slide 1
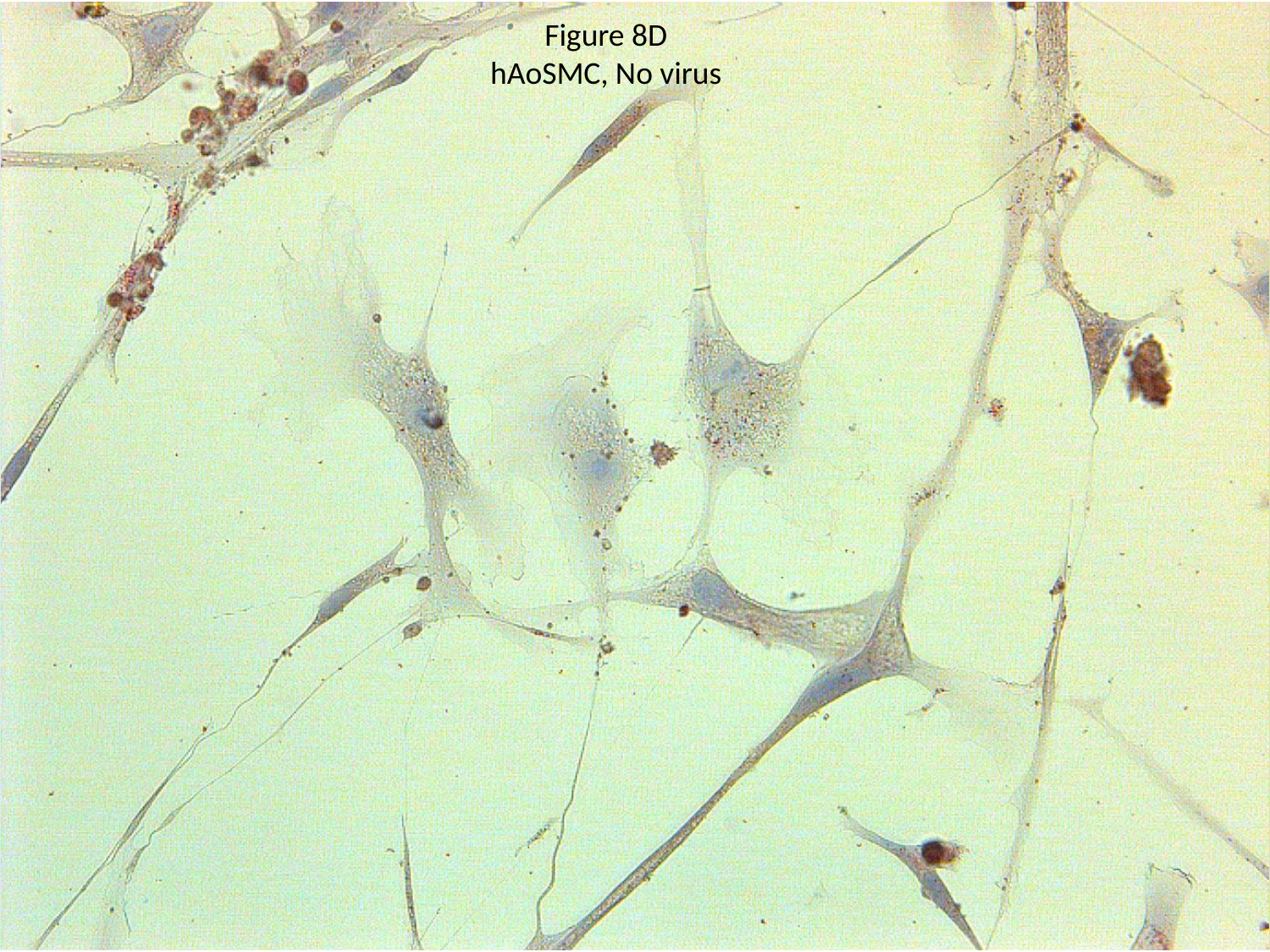

Figure 8D
hAoSMC, No virus

## Slide 2
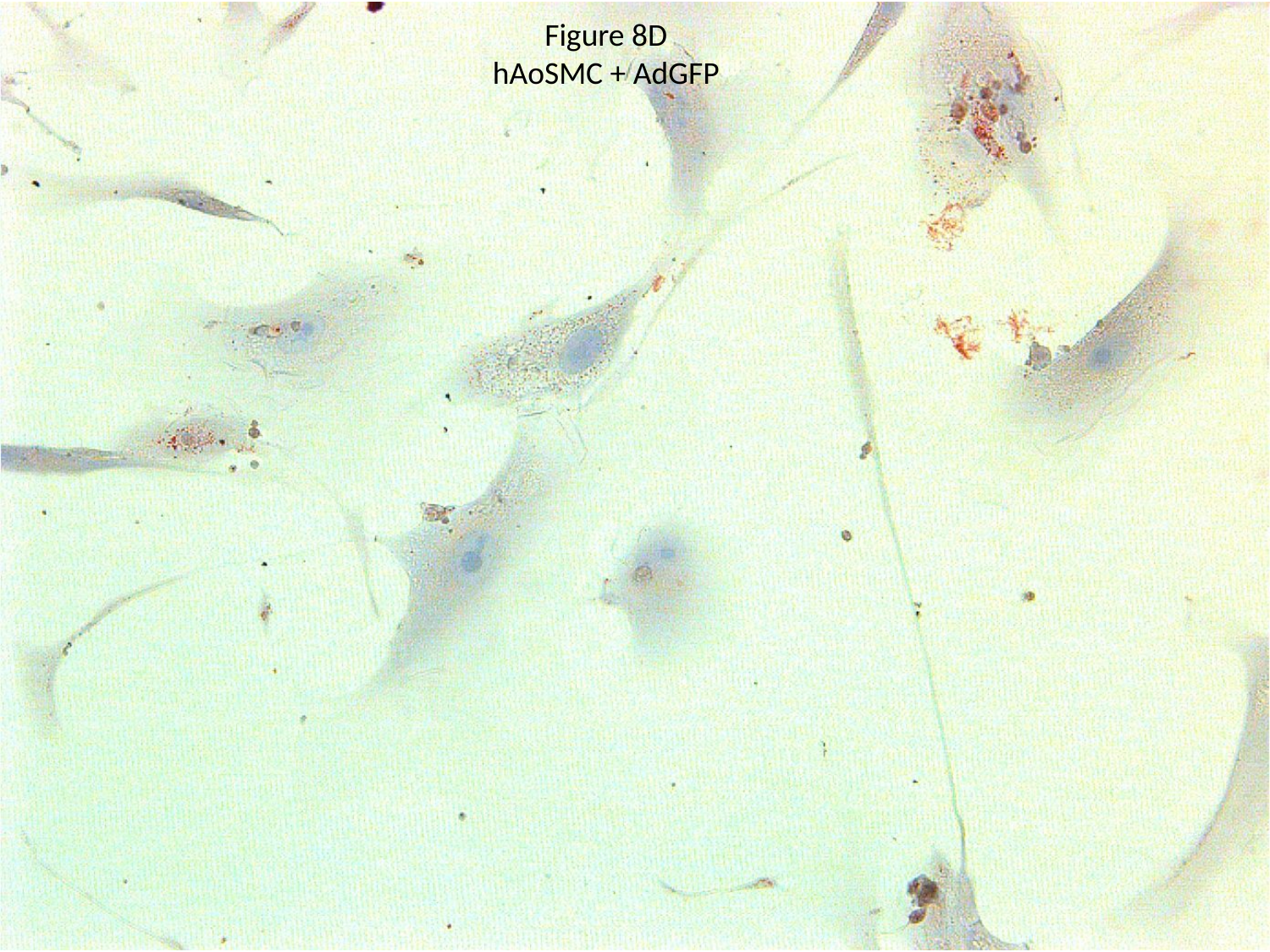

Figure 8D
hAoSMC + AdGFP

## Slide 3
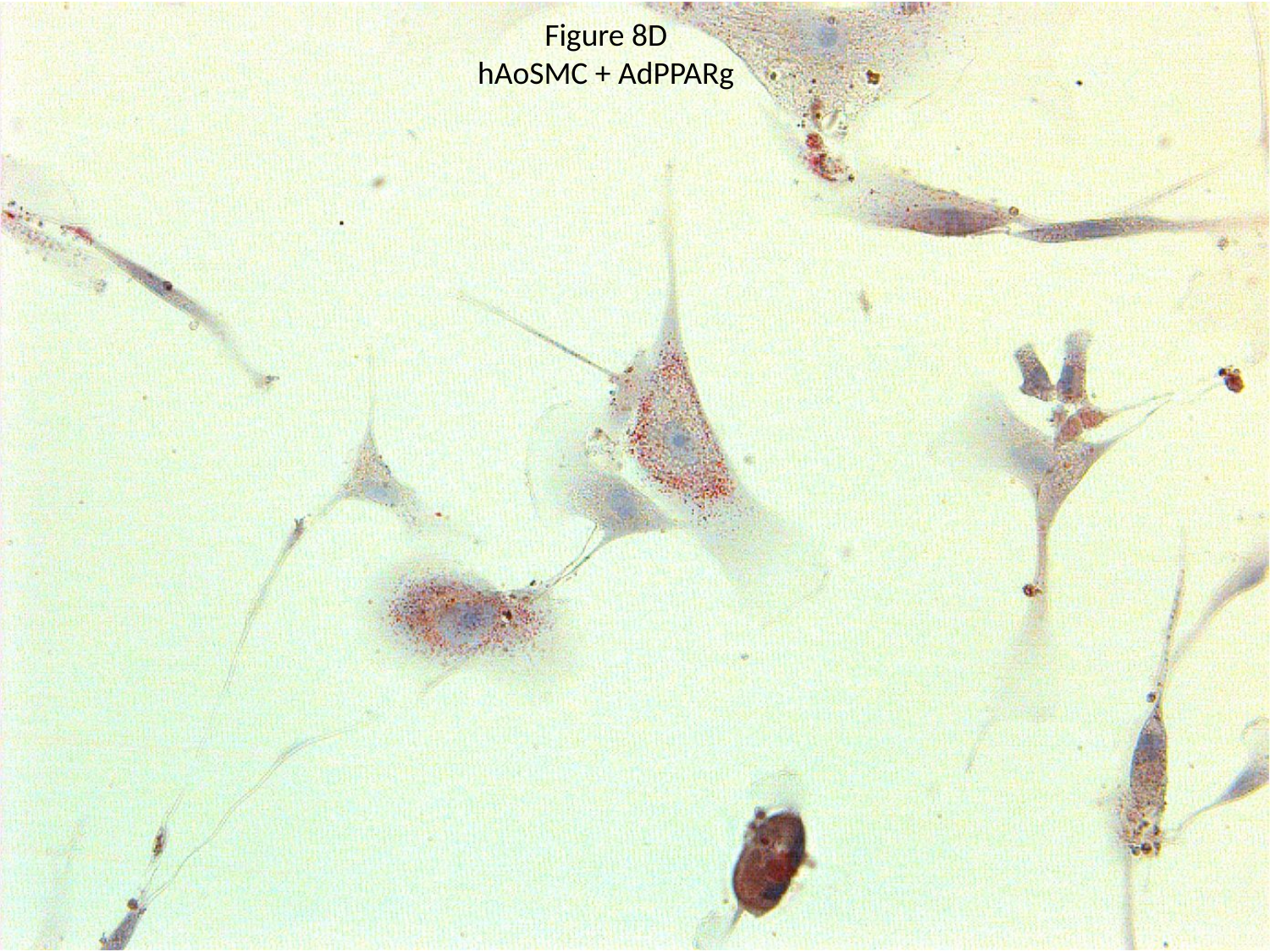

Figure 8D
hAoSMC + AdPPARg

Supplement: S8 Raw Data — (PPTX) [file pone.0139756.s020.pptx]
