# Supplementary material for: Smooth Muscle-Targeted Overexpression of Peroxisome Proliferator Activated Receptor-γ Disrupts Vascular Wall Structure and Function
Source: PLoS One. 2015 Oct 9;10(10):e0139756. doi: 10.1371/journal.pone.0139756 (PMC4599849; doi:10.1371/journal.pone.0139756)
Supplement: S7 Raw Data — (PPTX) [file pone.0139756.s019.pptx]

## Slide 1
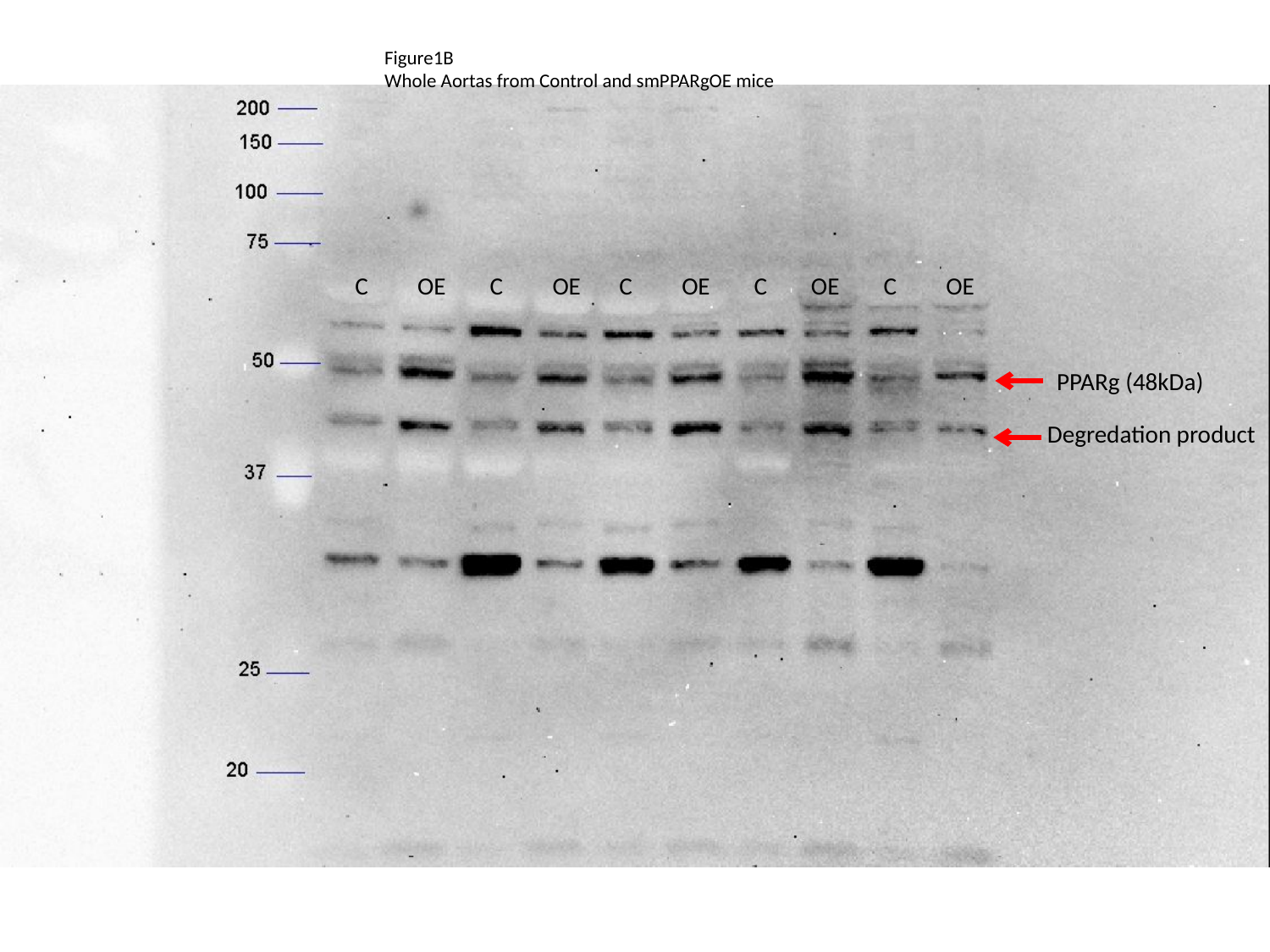

Figure1B
Whole Aortas from Control and smPPARgOE mice
C OE C OE C OE C OE C OE
PPARg (48kDa)
Degredation product

## Slide 2
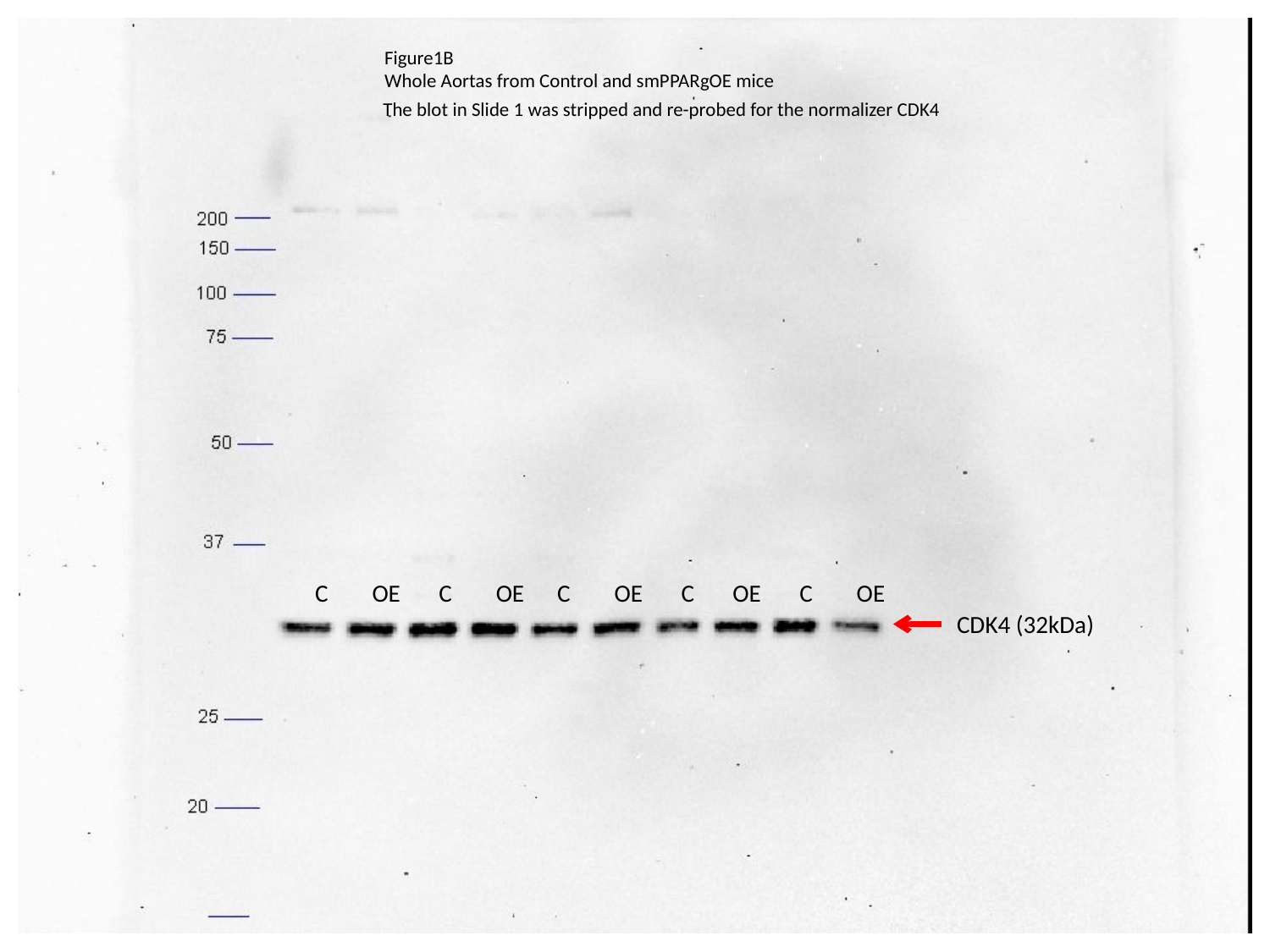

Figure1B
Whole Aortas from Control and smPPARgOE mice
The blot in Slide 1 was stripped and re-probed for the normalizer CDK4
C OE C OE C OE C OE C OE
CDK4 (32kDa)

## Slide 3
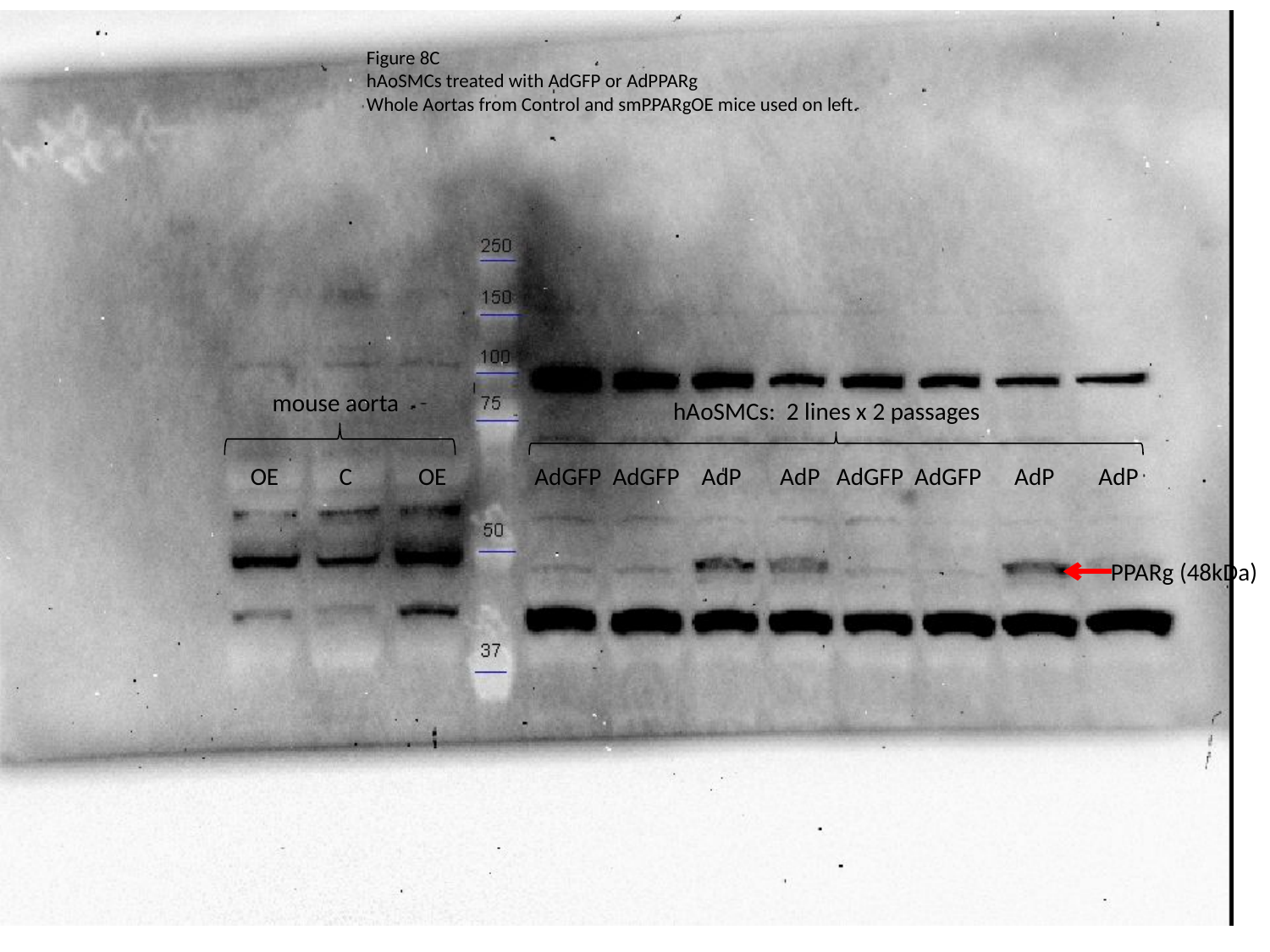

Figure 8C
hAoSMCs treated with AdGFP or AdPPARg
Whole Aortas from Control and smPPARgOE mice used on left.
mouse aorta
hAoSMCs: 2 lines x 2 passages
OE C OE AdGFP AdGFP AdP AdP AdGFP AdGFP AdP AdP
PPARg (48kDa)

## Slide 4
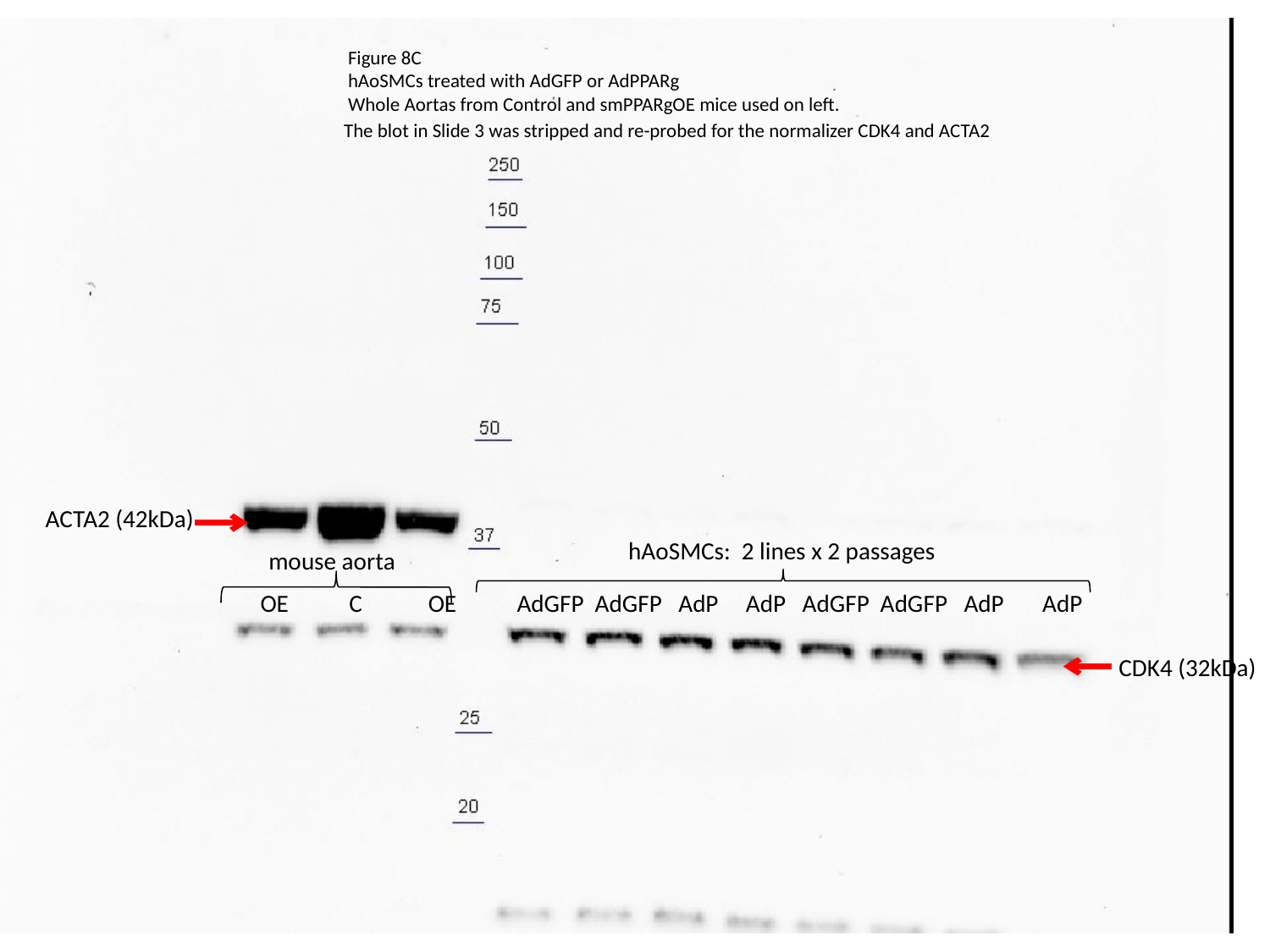

Figure 8C
hAoSMCs treated with AdGFP or AdPPARg
Whole Aortas from Control and smPPARgOE mice used on left.
The blot in Slide 3 was stripped and re-probed for the normalizer CDK4 and ACTA2
ACTA2 (42kDa)
hAoSMCs: 2 lines x 2 passages
mouse aorta
OE C OE AdGFP AdGFP AdP AdP AdGFP AdGFP AdP AdP
CDK4 (32kDa)
